# Comparative expression of soluble, active human kinases in specialized bacterial strains

**DOI:** 10.1371/journal.pone.0267226

**Published:** 2022-04-19

**Authors:** Allison Sunderhaus, Ramsha Imran, Elanzou Enoh, Adesola Adedeji, Taiye Obafemi, May H. Abdel Aziz

**Affiliations:** Department of Pharmaceutical Sciences and Health Outcomes, Fisch College of Pharmacy, The University of Texas at Tyler, Tyler, Texas, United States of America; Panjab University Chandigarh, INDIA

## Abstract

Kinases act as molecular switches for cellular functions and are involved in multiple human pathogeneses, most notably cancer. There is a continuous need for soluble and active kinases for *in-vitro* drug discovery and structural biology purposes. Kinases remain challenging to express using *Escherichia coli*, the most widely utilized host for heterologous expression. In this work, four bacterial strains, BL21 (DE3), BL21 (DE3) pLysS, Rosetta, and Arctic Express, were chosen for parallel expression trials along with BL21 (DE3) complemented with folding chaperones DnaJ/K and GroEL/ES to compare their performance in producing soluble and active human kinases. Three representative diverse kinases were studied, Epidermal Growth Factor Receptor kinase domain, Aurora Kinase A kinase domain, and Mitogen-activated protein Kinase Kinase. The genes encoding the kinases were subcloned into pET15b bacterial plasmid and transformed into the bacterial strains. Soluble kinase expression was tested using different IPTG concentrations (1–0.05 mM) at varying temperatures (37°C– 10°C) and induction times (3–24 hours). The optimum conditions for each kinase in all strains were then used for 1L large scale cultures from which each kinase was purified to compare yield, purity, oligomerization status, and activity. Although using specialized strains achieved improvements in yield and/or activity for the three kinases, none of the tested strains was universally superior, highlighting the individuality in kinase expression.

## Introduction

Kinases constitute one of the largest classes of druggable targets in humans due to their involvement in numerous cellular processes [1]. Their role in cell growth, division, and signal transduction is vital to maintain normal cellular homeostasis thus, their functional dysregulation is linked to multiple pathologies, most notably cancer [2]. There is a continuous need for purified kinases for structural, functional, and drug discovery purposes, yet the expression of active enzymes in readily available hosts remains an elusive task.

Several expression hosts are utilized to produce active kinases. While the baculovirus expression is the system of choice in insect cells [3], other systems like yeast were used successfully for expression of active human kinases [4]. *Escherichia coli* (*E*. *coli*) is a widely utilized host because it is inexpensive, easy to maintain, and usually has a high yield of proteins in a relatively short timeframe. However, past studies have shown that active kinases are rarely expressed in *E*. *coli* in a soluble form [5,6]. The solubility of enzymes is a key factor in assessing biological functions as it indicates proper folding, enzymes need to be in a soluble status to interact with their substrates, explaining the dependency of activity and protein–protein interactions on the degree of solubility [7]. Kinases are generally flexible proteins that are challenging to fold and often require post-translational modifications for activation that are unattainable with bacterial expression systems [8]. Despite the fact that 75% of human proteins, in general, can be expressed using *E*. *coli*, only 25% are actually active and soluble [9], the specific percentages for human kinases were not investigated but is probably lower.

Poor bacterial expression of kinases can stem from “leaky expression” when the genetic promoter of the expressing plasmids is not fully repressed, starting protein production in the host without induction [10]. Because kinases are usually toxic or restrict host cell growth resulting in a reduction of the cell biomass and consequently the yield of produced proteins, a drawback that can be minimized with tightly controlled expression [5]. Another cause for poor expression is that kinases expressed at a high rate under appropriate *E*. *coli* growth temperature (37°C) at which the internal bacterial folding chaperones cannot process the enzyme efficiently, resulting in misfolding and causing the proteins to become insoluble and trapped in inclusion bodies [5]. The folding of kinases was shown to improve through externally added chaperones [11], yet a recent study with detailed examination of the resulting proteins showed that most of the expressed kinases are in fact inactive soluble aggregates, highlighting the necessity of functional testing [6,12]. Makino, et al. discussed several specialized *E*. *coli* strains developed to overcome some of these limitations to improve protein expression in general [13].

Although several methods for successful expression of human kinases in *E*. *coli* were reported in literature, there are few comparative studies between these methods for the same kinase [8,14,15]. In this work, we compare the expression of three diverse enzymes: the tyrosine kinase domain of Epidermal Growth Factor Receptor (EGFR-KD), the serine-threonine kinase domain of Aurora A (AurKA-KD), and the full-length mixed kinase Mitogen-activated protein Kinase Kinase (MKK3). A recent study reported the bacterial expression of EGFR-KD using pCold plasmid that is auto-induced by low expression temperature [16]. The bacterial expression and purification of AurKA-KD and maltose-binding protein (MBP)-tagged MKK3 was previously reported [17–19], yet the stability of the full-length MKK3 was not tested after removal of the MBP tag, which limit its usefulness for some applications. The size of tags like MBP, N-utilization substance (NusA), and Glutathione-S-transferase (GST) makes them less desirable to use if structural studies are intended despite their efficiency in improving solubility and/or stability of expressed proteins.

We utilized the expression of the N-terminal His-tagged kinases in BL21 (DE3) *E*. *coli* strain for baseline comparison with four different expression improvement techniques: the addition of external folding chaperones and using the specialized strains: Rosetta, BL21 (DE3) pLysS, and Arctic Express. The same vector, pET15b, was used in all expression trials to prevent gene copy number and promoter type variations. Expression time, temperature, and induction times were optimized on a small scale following standard ranges [20–22], the optimum conditions were then used to prepare large-scale 1L expression followed by protein purification. Finally, the purified kinases were compared to assess the purification yield, purity, aggregation, and activity across the different tested strains/conditions.

## Materials and methods

### Chemicals and reagents

All chemicals were obtained from Fisher Scientific unless otherwise stated. LB/carbenicillin plates, Tris-Glycine-SDS, and TBST buffers were from Teknova. 4–12% Tris-Glycine gel, Unstained Protein Standards, and Trans-Blot Turbo Ready-To-Assemble (RTA) Mini 0.2 μm PVDF Transfer Kits for Western blots (WB) were from BioRad. The kinases were probed with anti-His Tag Antibody HRP-labeled Mouse Monoclonal IgG (R&D Systems # MAB050H).

### Kinase constructs in bacterial plasmids

AurKA-KD in pET15-b vector (Uniprot accession number O14965-1, residues 123–403) was used for expression, and the same vector was used to subclone EGFR-KD and MKK3 from their respective vectors. EGFR-KD (Uniprot accession number P00533-1, residues 682–1022) was amplified from pFastBac vector using sense primer 5’-CGGTCCGAGCTCATGTCGTAC-3’, anti-sense primer 5’-CTTCTCGAGAAGCTTTCAG-3’ and subcloned into the *SacI/XhoI* linearized pET15-b vector. The vector was then modified to add a *NdeI* site upstream of AurKA-KD gene using sense primer 5’-GCAGCGGCCATATGAACAAAGAAATTTTG-3’, anti-sense primer 5’-CTTTGTTCATATGGCCGCTGCTGTGATGATG-3’. MKK3 (Uniprot accession number P46734-1, residues 1–347) was amplified from pICZ-α plasmid using sense primer 5’-CGACCGAACATATGTATTTTCAGGGCATG-3’, anti-sense primer 5’-GAATTCCTGCTAGCCCGGGTC-3’ and subcloned into the *NdeI/BmtI* linearized modified pET15-b vector. All constructs were transformed into Top10 F’ cells for plasmid amplification and verified by sequencing before starting the expression trials.

### Small-scale optimization of expression

The plasmids were individually transformed into four *E*. *coli* strains BL21 (DE3), BL21 (DE3) pLysS, Arctic Express, and Rosetta (S1 Table). When expression in BL21 (DE3) complemented with folding chaperone was tried, transformation with plasmids pGro7 and pKJE7 containing groES/EL and dnaK/J/E chaperones (Takara cat no. 3340) was performed initially followed by positive transformants selection and subsequent transformation with the kinases’ plasmids per manufacture’s recommendations. Transformation mixtures were incubated overnight at 37°C on Lysogeny broth (LB) plates supplemented with appropriate selection antibiotic(s) (S1 Table). Positive transformants were cultured in Terrific broth (TB) supplemented with the respective antibiotic(s) at 37°C until they reached the mid-log growth phase (OD 0.6). For BL21 (DE3) complemented with chaperones, 0.5 mg/ml arabinose was added to the culture to induce the expression of the folding chaperones. Cultures were then divided into 5 ml aliquots, cooled to the tested induction temperatures, and IPTG added at 0.05, 0.5, or 1 mM. Samples were collected for analysis of soluble protein at different time intervals. Collected cell samples were lysed in 1 ml of B-PER^™^ Complete reagent supplemented with 300 mM NaCl, 10 mM imidazole, and protease inhibitors and processed per manufacturer’s recommendations. Pellets were treated with 8M urea, and all samples were analyzed by SDS-PAGE to determine optimal expression conditions for each kinase in the compared conditions and strains.

### Large scale expression and purification

Optimized conditions (temperature, IPTG concentration, and induction time) for each kinase in all tested strains/conditions were used for preparing large-scale 1L cultures in TB supplemented with appropriate antibiotics from freshly transformed cells. The cultures were harvested by centrifugation at 4,000 g for 15 min at 4°C. Collected pellets were washed with PBS and stored at -80°C until purified; all the following work was carried out at 4°C. The pelleted cells were resuspended in lysis buffer (25mM Tris HCl pH 8, 1mM TCEP, 10mM NaCl, 10mM Imidazole, 5% Glycerol, 0.01% Tween) supplemented with protease inhibitor tablet and lysed using a Microfluidizer with two to three passes under 15,000 psi. The lysates were clarified by centrifugation at 20,000 rpm for one hour at 4°C, then loaded onto a BioRad FPLC purification system equipped with an equilibrated 1ml His Trap FF column (GE Healthcare Life Sciences). The column was washed, and the protein was eluted with an elution buffer gradient (25mM Tris HCl pH 8, 1mM TCEP, 10mM NaCl, 250mM Imidazole, 5% Glycerol, 0.01% Tween). The eluent was pooled and loaded onto an equilibrated Superdex 75 Increase 10/300 GL (GE Healthcare Life Sciences) size exclusion column and eluted using a buffer of 50 mM Tris HCl pH 8, 150 mM NaCl, and 0.25 mM TCEP. The molecular weights of the protein peaks were estimated using a calibration curve constructed with standard protein samples from the Gel Filtration LMW calibration Kit (GE Healthcare Life Sciences) prepared per manufacture’s protocol and analyzed under the same conditions as the studied kinases per published protocols [23].

Collected fractions were concentrated using washed Amicon Ultra Centrifugation filters (10 kDa molecular weight cutoff) and protein concentrations determined using 660 nm protein assay (Pierce) with bovine serum albumin as the reference standard. The purification yield in mg purified protein/L culture was calculated and purity was assessed using SDS-PAGE either using Mini-PROTEAN TGX regular or stain-free gels (BioRad) or Amido Black post-transfer stain after transferring to a PVDF membrane. PageRuler^®^, EZ Prestained, or Unstained Protein Standards were used to ascertain the molecular weight of the purified kinases. WBs were carried out on a GoBlot system (Cytoskeleton) by blocking the membrane for one hour in 5% Bovine Serum Albumin solution (BSA) in TBST, then incubating for one hour with the primary antibody (1:1000 dilution). Chemiluminescence detection was done using Thermo Scientific^™^ Pierce^™^ ECL Western Blotting Substrate per the manufacturer’s protocol.

### Assessment of kinase activity

The enzymatic activity of the purified kinases was tested using a kinetic coupled assay that measures the rate of ATP consumption per published protocols [24,25]. Briefly, the kinase reaction and conversion of ATP to ADP is coupled to a pyruvate kinase/lactate dehydrogenase (PK/LDH) system that converts NADH to NAD^+^ where the signal monitored was the decrease in NADH absorbance at 340 nm. The reaction mixture contained 20 mM Tris, pH 8, 2 mM ATP, 10 mM MgCl_2_, 55 U/ml PK/LDH mix (Sigma), 1 mM phosphoenolpyruvate and 0.3 mg/ml NADH. Substrates for each kinase were added to the reaction mixture as follows: poly-4Glu:Tyr peptide for EGFR-KD, dephosphorylated myelin basic protein for AurKA-KD, and p38 γ for MKK3 (final concentrations of 1 mg/ml, 4 μg/ml, and 16 μg/ml; respectively). The reaction rate was monitored over 15 min, and the early linear slope of the reaction (theoretical 10% substrate consumption) was normalized against blank reactions that contained all the reaction mixture components except for the kinase. The slopes were mathematically transformed to NADH concentration using NADH extinction coefficient (6220 L mol^-1^ cm^-1^). The enzyme activity was normalized to obtain the reaction rates using the rate of product formation in μM/sec per μM enzyme used in the reaction mixture per published protocols [24].

## Results and discussion

### Small changes in induction conditions significantly impact the kinases expression

Changes in induction conditions (temperature, inducer concentration, and time) are long known to impact protein expression in the *E*. *coli* [26]. Kinases are more prone to these changes given how challenging their heterologous expression is, especially in bacterial host systems [12]. To assess the expression of the studied kinases using the five expression strains/conditions, we tested three levels of IPTG concentrations (0.05, 0.5, and 1 mM) under different induction temperatures (10–37°C) and times (3 hours or ON: overnight) as recommended for each strain. The inducer concentration with highest expression level under the different induction temperatures and times for all strains is shown in S2 Table. As expected, a wide variation in soluble expression levels was noticed; the optimized expression conditions in each strain for the three kinases are summarized in Table 1. SDS-PAGE gels showing expression trials are freely available at Mendeley Dataset at http://dx.doi.org/10.17632/2w3ckb8knw.1#file-56504d8b-d138-42ac-980c-223fa1e63bb6.

**Table 1:** The optimized expression conditions, yield, and activity for the expressed kinases in different bacterial strains (all strains showed optimum expression with ON induction time). The reaction rates were calculated as detailed in Material and Methods section and represented in Fig 6.

	Temperature (°C)	IPTG (mM)	Yield (mg/L)	Rate of Reaction (×10^−3^ sec^-1^)
**EGFR-KD**				
BL21	25	0.05	1.42	9.54 ± 1.89
BL21+Chap.	25	1	1.87	14.89 ± 0.31
BL21 pLysS	25	1	1.25	10.29 ± 2.10
Arctic Express	15	0.05	ND*	ND
Rosetta	25	0.05	2.2	23.22 ± 2.86
**AurKa-KD**				
BL21	25	0.05	2.48	27.00 ± 5.03
BL21+Chap.	25	0.5	3.38	79.76 ± 5.06
BL21 pLysS	18	0.05	9.53	73.76 ± 2.62
Arctic Express	10	0.05	ND	ND
Rosetta	25	1	0.56	8.99 ± 1.09
**MKK3**				
BL21	25	1	1.44	6.67 ± 0.02
BL21+Chap.	25	0.05	0.73	31.08 ± 0.65
BL21 pLysS	25	1	1.32	7.91 ± 0.29
Arctic Express	10	1	ND	ND
Rosetta	18	1	0.58	50.23 ± 6.05

*ND: Not determined (yield was < 0.2 mg/ml).

**Fig 1:**
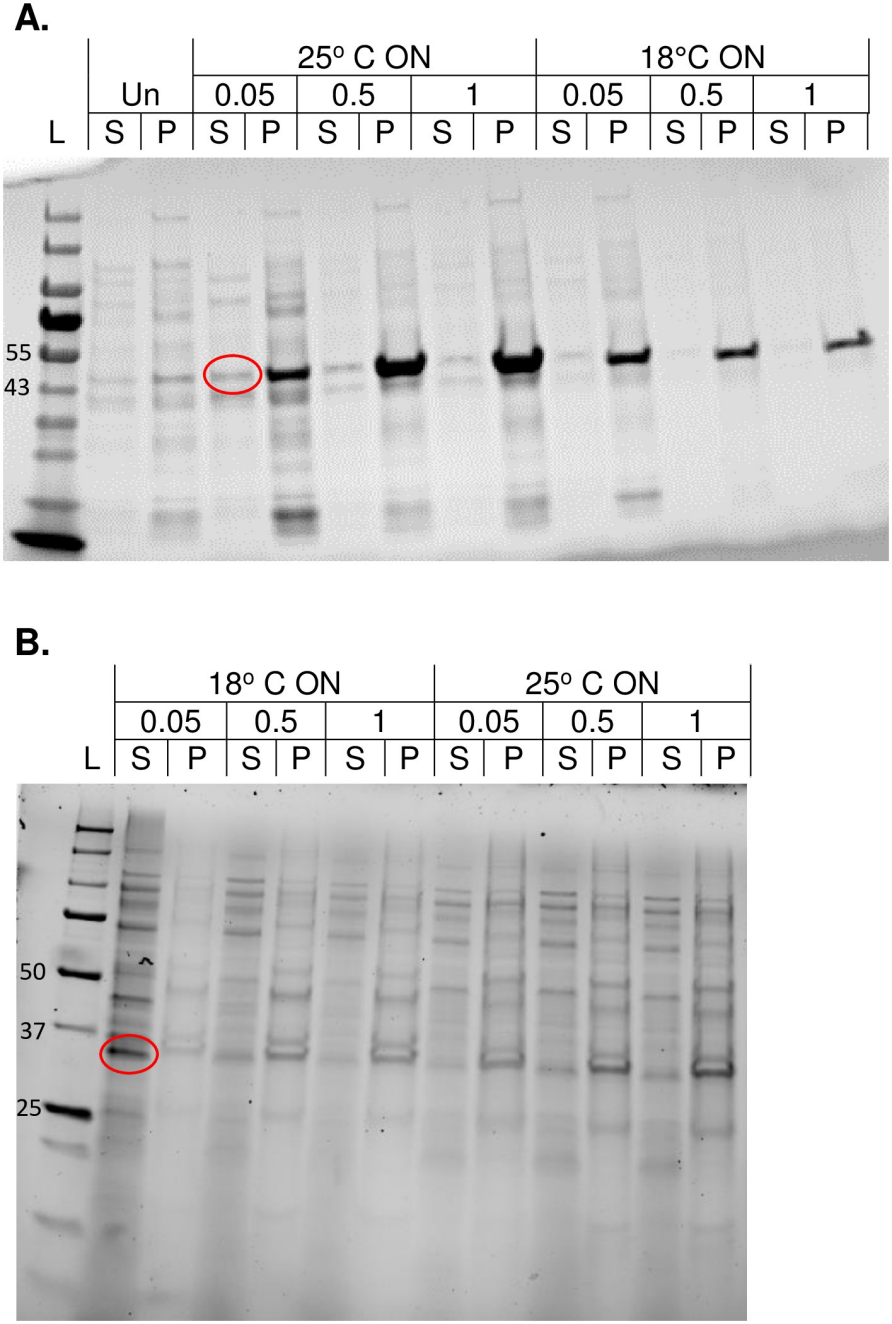
SDS-PAGE gels showing the effect of induction concentration and temperature on expression. A) EGFR-KD expression in BL21, and B) AurKA-KD expression in BL21 (DE3) pLysS strains with a similar trend of increasing soluble expression with lower IPTG concentrations and opposite expression trends with temperature of induction (Raw gels are shown in S1 Fig). L: Molecular weight ladder, Un: Uninduced sample, S: Supernatant and P: Pellet of cell lysates, ON: Overnight expression, and the numbers represent the concentrations of IPTG in mM.

Highlighted in Fig 1A is EGFR-KD expression in BL21 (DE3), it is shown that, for the same induction time (ON), a change of the induction temperature from 25°C to 18°C resulted in loss of the soluble expression of the protein. The opposite effect is noted in Fig 1B for AurKA-KD expression in BL21 (DE3) pLysS strain, where the lower temperature of induction led to a marked increase in soluble expression (raw gels in S1 Fig). The effect of the inducer concentration is also apparent for both proteins, where a gradual increase in EGFR-KD soluble expression occurs with lower IPTG concentration and a marked increase in AurKA-KD expression only at 0.05 mM IPTG.

**Fig 2:**
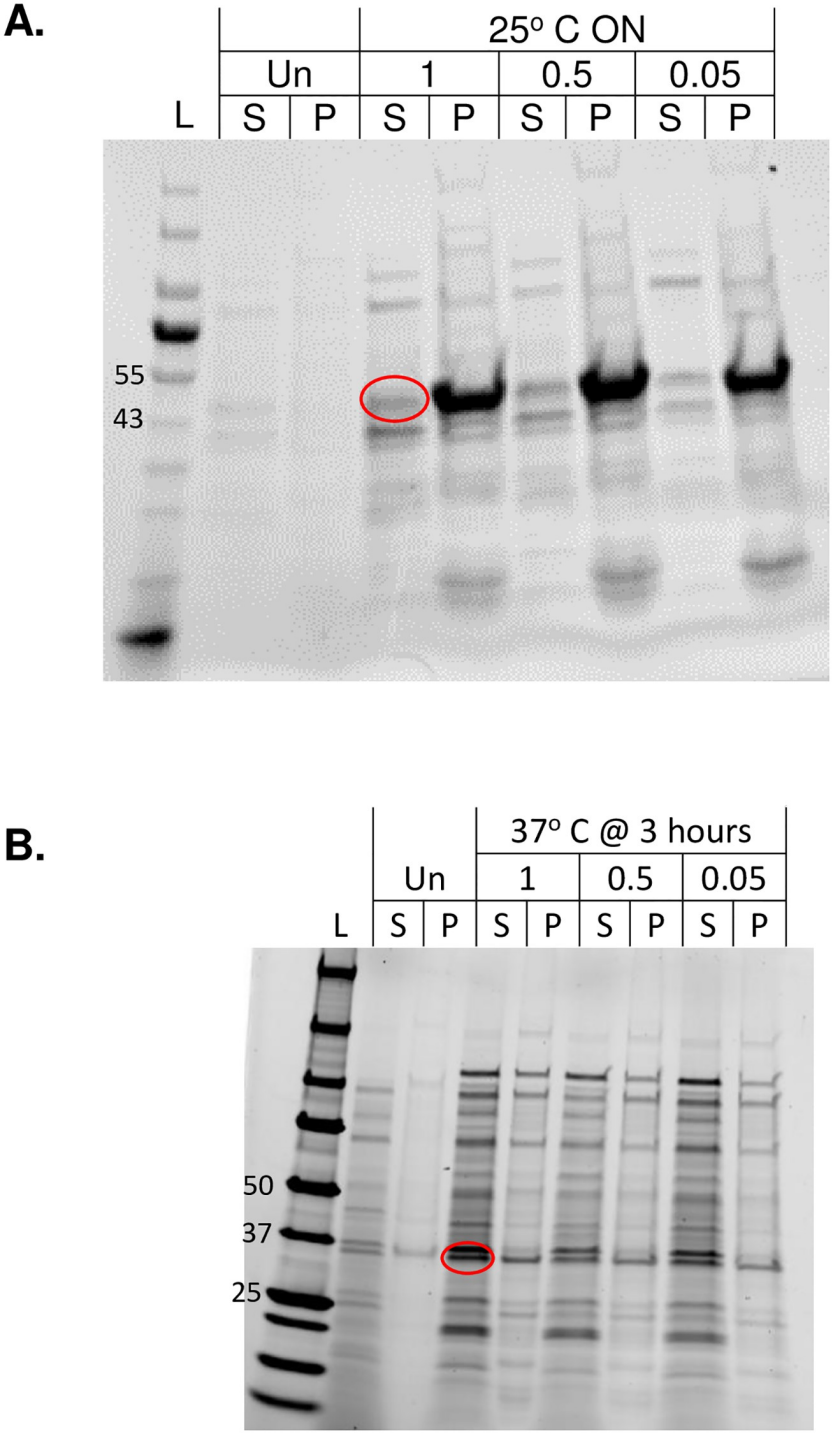
SDS-PAGE gels showing the effect of adding folding chaperones on expression. A) EGFR-KD and B) AurKA-KD expression in BL21 (DE3) complemented with folding chaperones showing a similar trend of equal soluble expression at different IPTG concentrations. (Raw gels are shown in S2 Fig, and letter abbreviations are the same as Fig 1).

When added chaperones were used to improve the expression in the BL21 (DE3) strain, it was noted for both proteins that there is an increase in soluble protein expressed and that all IPTG concentrations showed almost equal expression (Fig 2; raw gels in S2 Fig). The difference BL21 (DE3) expression of EGFR-KD in the absence and presence of added chaperones at the same induction time and temperature can be compared in Figs 1A and 2A. The results can be attributed to the ability of the additional folding chaperones to maximize soluble expression even at high IPTG concentrations that were shown to promote otherwise misfolding.

MKK3 showed a low expression level in most tested strains/conditions where expression was either not detected at the expected molecular weight or showed equally weak expression under all tested conditions (S2 Table). Because of the low expression, it becomes difficult to visually determine the optimum conditions for expression across different SDS-PAGE gels; thus, the favorable conditions in some of the strains were confirmed by running the soluble fractions on the same gel followed by a WB of some of the samples of confirmed expression with anti-His antibody to verify identity (S3 Fig).

### Specialized strains show variable yields with expected oligomerization patterns

**Fig 3:**
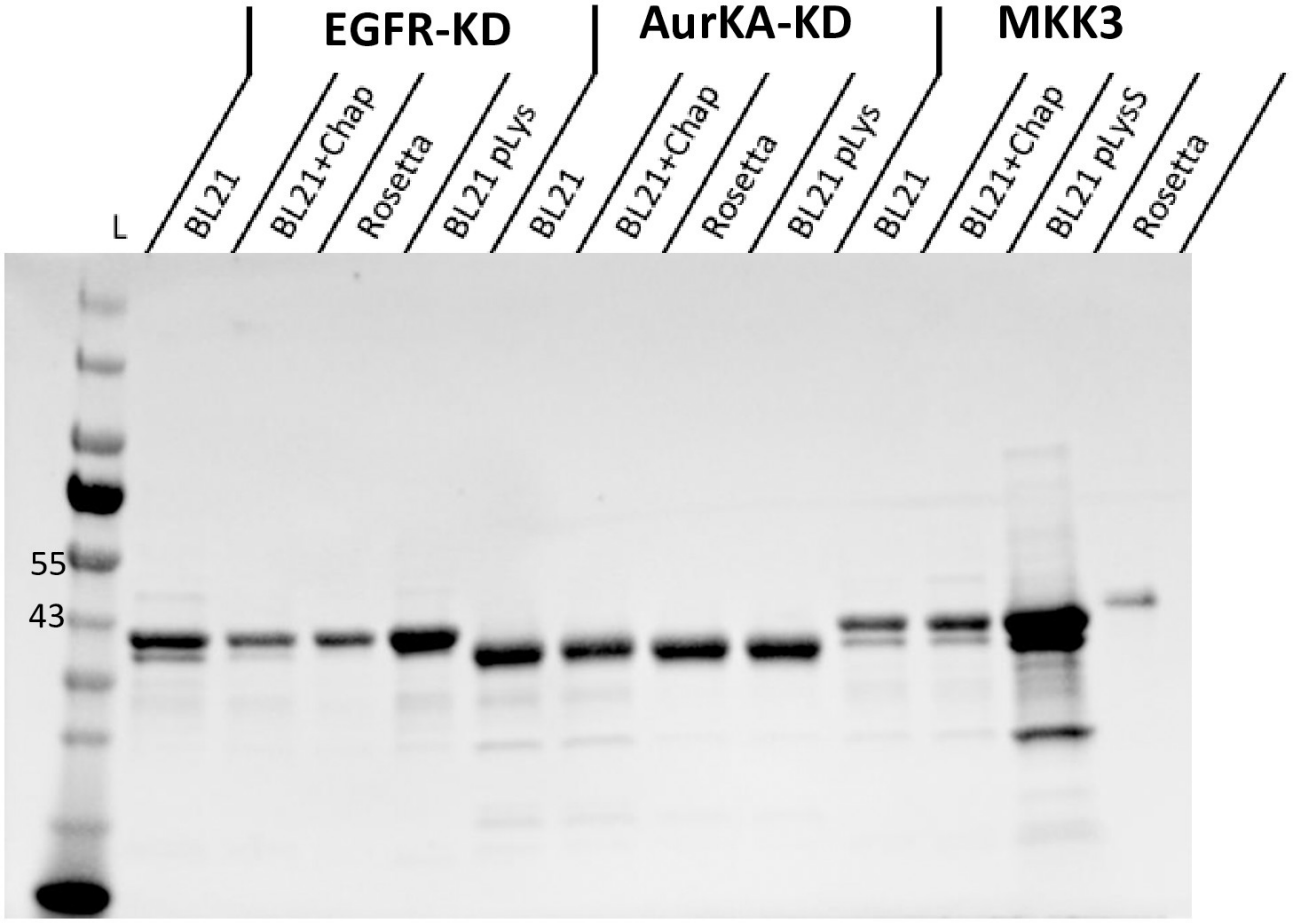
SDS-PAGE showing the final purified proteins. Proteins were loaded at 0.2–1.3 mg/ml final concentration (Raw gel in S4 Fig).

The optimized conditions for expression of the kinases in the tested strains were used for protein production in 1L cultures to compare the characteristics of the produced kinases. Analysis of native state molecular weights and oligomerization was carried out by size exclusion chromatography (SEC) as detailed in the Materials and Methods section. Kinase domains are often expressed in multiple oligomerization forms, most commonly as a mixture in equilibrium between monomer and dimer form [27]. The oligomerization status may affect the homogeneity and activity of the expressed kinases and allow for accurate characterization, especially for structural studies. MKK3 showed moderate aggregation levels in BL21 (DE3) with and without added chaperones, while EGFR-KD and AurKA-KD had minimal aggregation under the tested expression conditions. The aggregation of MKK3 with BL21 (DE3) with and without added chaperons may suggest that the reason for aggregation cannot be resolved by directly improving the folding efficiency. The oligomerization status improved by alleviating the effect of the toxic nature of the kinase on its host with the BL21 pLysS strain or by improving the translation through using Rosetta strain. All proteins were concentrated to 0.3–1.3 mg/ml for further testing and were of high purity ≥ 90% (Figs 3 and S4, SDS-PAGE gels of HisTrap purifications are available in the Mendeley Dataset at http://dx.doi.org/10.17632/2w3ckb8knw.1#file-56504d8b-d138-42ac-980c-223fa1e63bb6).

**Fig 4:**
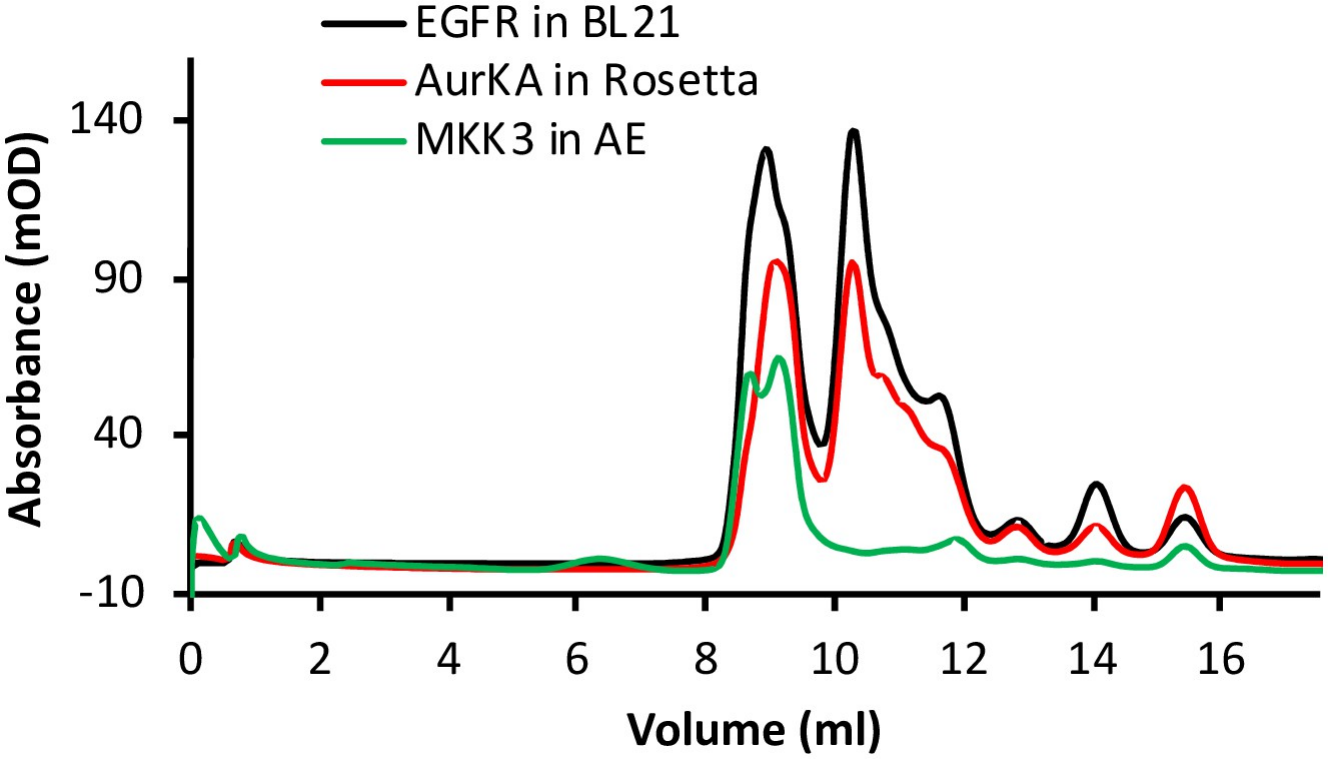
Representative Chromatograms showing oligomerization pattern of the expressed kinases. The profile shows the eluent of the Superdex 75 Increase 10/300 GL gel filtration column. (SEC profiles for all kinases are shown in S5 Fig).

SEC profiles show that the kinases expressed in the Arctic Express strain has a major peak representing high molecular weight proteins that were also seen in SDS-PAGE gels. In contrast, the three kinases expressed in all other strains are purified as a mixture of monomeric and dimeric forms as previously reported with insect cells and yeast expression (Figs 4 and S5) [23,27]. Further characterization was done using Dynamic Light Scattering to determine the average particle size and dispersity. The size distribution plots revealed that the purified EGFR for example has a size comparable to the same construct expressed from insect cells. The polydispersity index calculated for all samples show that the enzymes are homogenous and monodispersed in nature (S6 Fig and S3 Table). The absence of the tested kinases’ expression in the Arctic Express strain highlights how small-scale expression may not translate to large-scale protein production. A recent attempt to express EGFR kinase domain using pColdI vector and low-temperature induction was successful, underlining that some cold induction with specialized vectors may have a different expression outcome than specialized strains with a low growth temperature [16].

**Fig 5:**
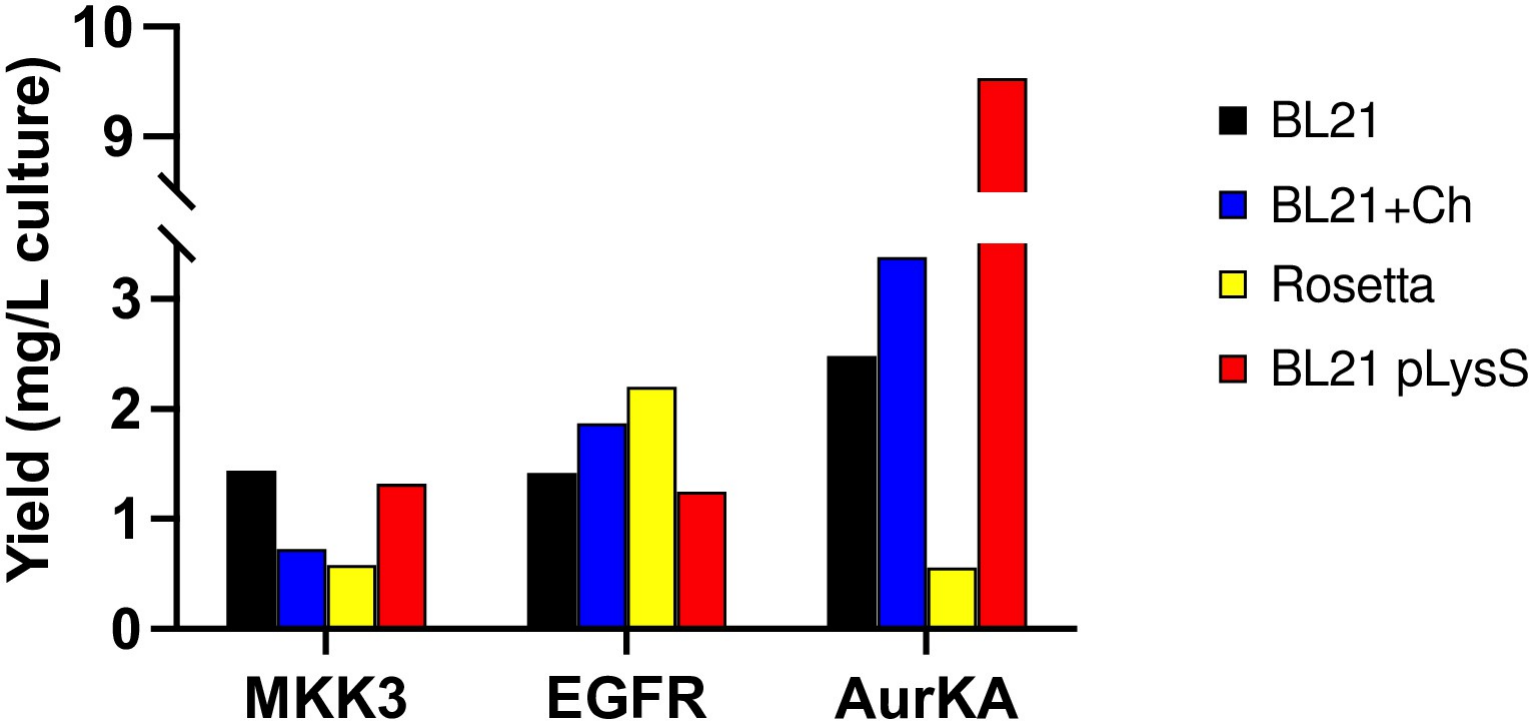
Purification yield of the expressed kinases. The yield is expressed as mg purified protein per 1L bacterial culture in the different expression strains/conditions. Protein concentrations were calculated as detailed in the Material and Methods section.

A 3-fold increase in AurKA-KD purification yield was noticed in BL21 (DE3) (with and without added folding chaperones) compared to Rosetta strains; an additional 3-fold increase was obtained with BL21 (DE3) pLysS strain (Fig 5). The expression yields obtained in the tested strains were comparable with an average of 1.69 mg/L culture for EGFR-KD and 1.02 mg/L culture for MKK3 (Table 1).

### Detected kinase yields do not necessarily correlate with enzymatic activity levels

**Fig 6:**
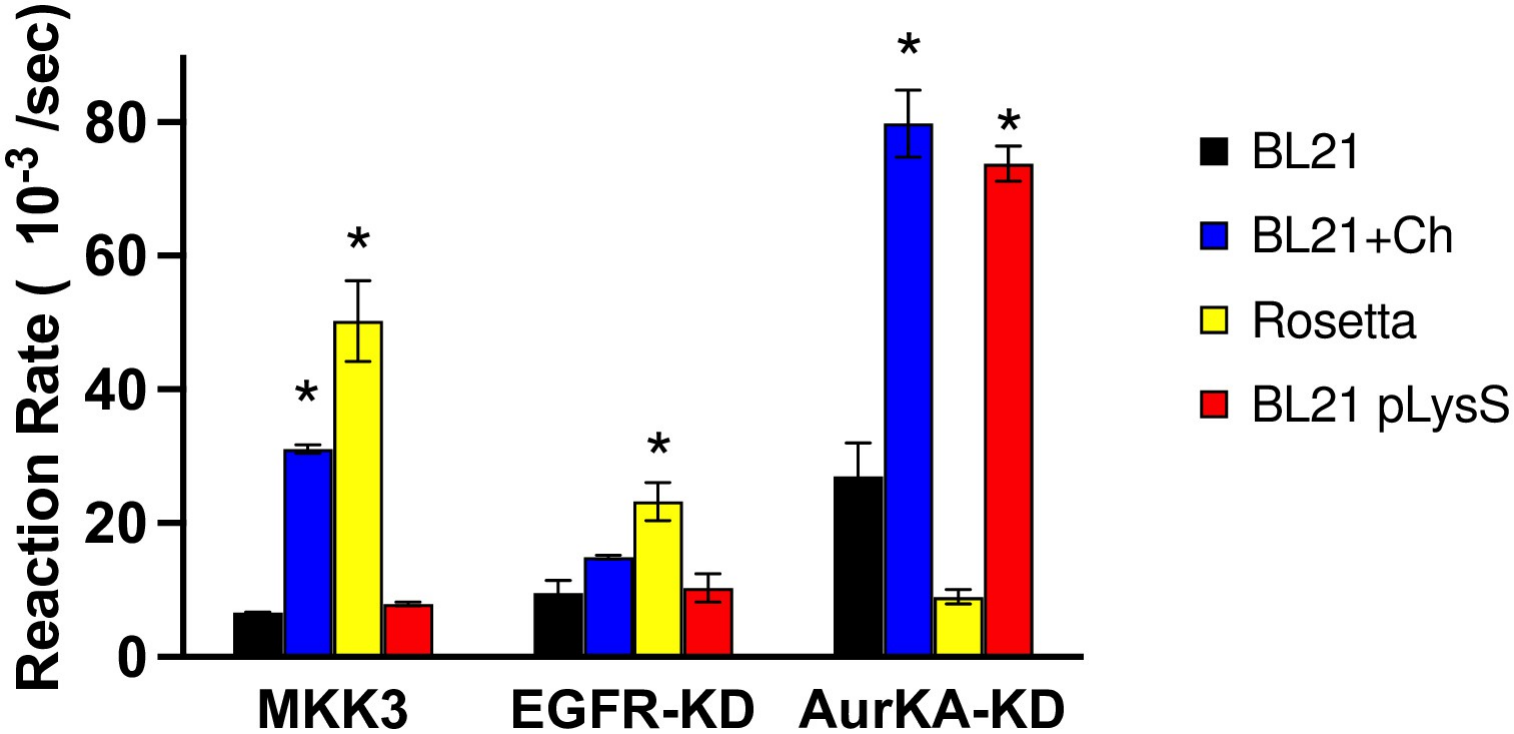
Activity of the purified kinases. The phosphorylation reaction rate for the purified enzymes in the different expression strains/conditions using the coupled kinase assay as detailed in the Materials and Methods section.

The coupled assay was used to assess the activity of all kinases where the activity of the kinases expressed in BL21 (DE3) was used as a baseline for statistical comparison to the utilized strains/conditions. The results summarized in Fig 6 and Table 1 show a significant increase in the enzymatic activity when EGFR-KD is expressed in Rosetta strain, and MKK3 expressed in either Rosetta or BL21 (DE3) with added chaperones, even though the yields of total protein obtained from expression was lower or comparable to other strains. Notable in MKK3, despite the presence of aggregated forms of the protein in BL21 with and without chaperones. The yield was lower when chaperones were added, yet the improved folding achieved with the chaperones has significantly improved the activity of the expressed protein. AurKA-KD showed a significant increase in activity when expressed in either BL21 (DE3) with added chaperones or BL21 (DE3) pLysS. It was interesting to see that, similar to MKK3, adding the chaperone significantly increased the activity even though the protein yields were comparable to BL21 (DE3) and 3-fold lower than BL21 (DE3) pLysS. The specific activity of the enzymes was compared to the reported values of the same constructs prepared in either sf9 insect cells of *Pichia pastoris* hosts or to commercially available enzymes as applicable (S4 Table). Of note that oftentimes, the enzyme’s activity is affected by concentration due to the dependency of the activity on the aggregation status [28], thus we ensured that for each kinase the activity was tested at the same concentration level across the different hosts. Brignola, et al. has determined that while the enzymatic activity of EGFR expressed in insect cells was stable over the course of one-hour, other human kinases lost up to 10% of their activity within 10 minutes [29]. We monitored the activity of the expressed constructs over 2–4 hours at variable temperatures (4, room temperature, and 37°C). While EGFR-KD maintained 71–97% of its activity after 4 hours, MKK3 had 55–65% activity after only 2 hours. AurKA-KD showed a temperature dependence stability, while stable at 4°C and room temperature, only 32% of the activity was maintained at 37°C (S7 Fig). These results highlight the individuality of human kinases’ behavior in solution and the importance of unbiased investigation of each construct behavior.

## Conclusion

We have analyzed and compared the expression of EGFR-KD, AurKA-KD, and MKK3 in specialized bacterial strains to determine which bacterial system and conditions produced soluble and active protein. Protein expression was successful for all three kinases with variable yield, activity, and solubility. Some bacterial strains, IPTG concentrations, or induction temperatures seem to improve expression as expected. The three kinases displayed variable expression levels, protein yields, and activity in different tested strains with little correlation between soluble protein yield and activity. The results highlight the need for scrutiny of expression conditions and strain testing. Screening may initially indicate success in expressing the target kinase in high yields. Yet, final selection should also include analysis of aggregation and functional activity to ascertain that the expressed proteins are correctly folded and are indeed functional. The purified kinases can be used for functional assays and for drug discovery purposes investigating possible inhibitors that can be used as anticancer agents.

## Supporting information

S1 Fig Raw SDS-PAGE gels showing effect of induction concentration and temperature on expression. A, B) Raw SDS-PAGE for gels shown in Fig 1A and 1B; respectively, letter abbreviations are the same as in Fig 1 in text.
(PDF)

S2 Fig Raw SDS-PAGE gels showing the effect of adding folding chaperones on expression. A, B) Raw SDS-PAGE for gels shown in Fig 2A and 2B; respectively, letter abbreviations are the same as in Fig 1 in text.
(PDF)

S3 Fig Expression conditions for MKK3. A. Raw SDS-PAGE gel showing MKK3 expression in different strains and conditions that were difficult to compare across different initial expression gels, and B) Western blot with anti-His antibody for some of the samples to confirm protein identity, 2X refers to two-fold dilution of some samples to prevent overexposure and other letter abbreviations are the same as in Fig 1 in text.
(PDF)

S4 Fig Raw SDS-PAGE showing the final purified proteins. Raw image for gel shown in Fig 3 in text.
(PDF)

S5 Fig SEC Chromatograms of A) EGFR-KD, B) AurKA-KD, and C) MKK3 in all strains. The inset show the twin peaks profile observed in the SEC profiles of the same constructs prepared in SF9 insect cells for EGFR-KD separated on the same SEC coumn (reproduced with permission from reference 19, Fig 4), and *Pichia pastoris* for AurKA-KD and MKK3 separated on HiLoad 16/600 Superdex 200 pg (SPS-PAGE for the three shown profiles can be found in references 4 and 19).
(PDF)

S6 Fig Size distribution plot for EGFR-KD. The particle size profiles from samples expressed in the reported bacterial strains are compared to the same construct prepared in sf9 insect cells and characterized in reference 19.
(PDF)

S7 Fig Stability of the purified kinases at different temperatures. The enzymes were stored at the indicated temperature and the activity was measured at the indicated time points as detailed in the Materials section.
(PDF)

S1 Table *E*. *coli* strains used in the study. The antibiotics used in culture were ampicillin at 100 μg/ml as the selection marker for the pET-15b plasmid used to host the kinases, in addition to the pre-existing resistance marker for each strain, if applicable.
(PDF)

S2 Table The inducer concentration with the highest expression level under different induction temperatures and times. The optimum conditions chosen for large-scale protein expression for the kinases in each bacterial strain are highlighted in bold (summarized in Table 1 in text).
(PDF)

S3 Table The average particle size diameter (d) and Polydispersity index (PDI) of the purified kinases. Samples were assessed using dynamic light scattering as mentioned in Discussion section. PDI was calculated by dividing the square of the standard deviation of the protein peak by the square of its average diameter.
(PDF)

S4 Table The specific activity for each purified kinase. The activity was calculated by converting the rate of substrate formation in μmol/sec to nmol/min/mg enzyme and compared to the specific activity of commercially available active enzymes and/or the published results of the same constructs prepared in insect cells for EGFR-KD (reference 19 in text) or *Pichia pastoris* for AurKA-KD and MKK3 (reference 4 in text).
(PDF)

S1 File (PDF)
