# Supplementary material for: Comparative expression of soluble, active human kinases in specialized bacterial strains
Source: PLoS One. 2022 Apr 19;17(4):e0267226. doi: 10.1371/journal.pone.0267226 (PMC9017934; doi:10.1371/journal.pone.0267226)

S1 Fig. Raw SDS-PAGE gels showing effect of induction concentration and temperature on expression A, B) Raw SDS-PAGE for gels shown in Figure 1A and B; respectively, letter abbreviations are the same as in Figure 1 in text.

A.

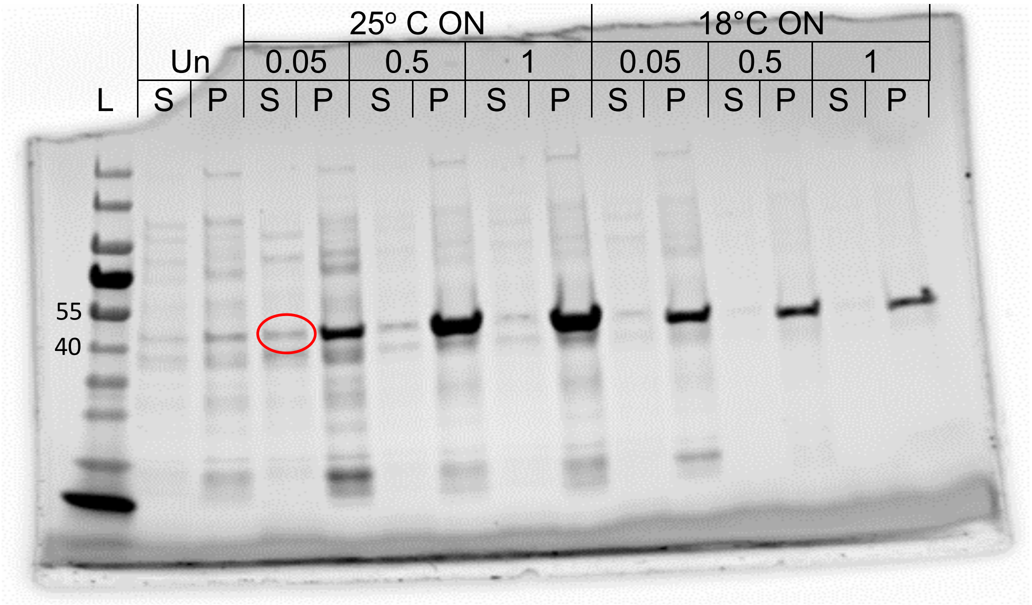

B.

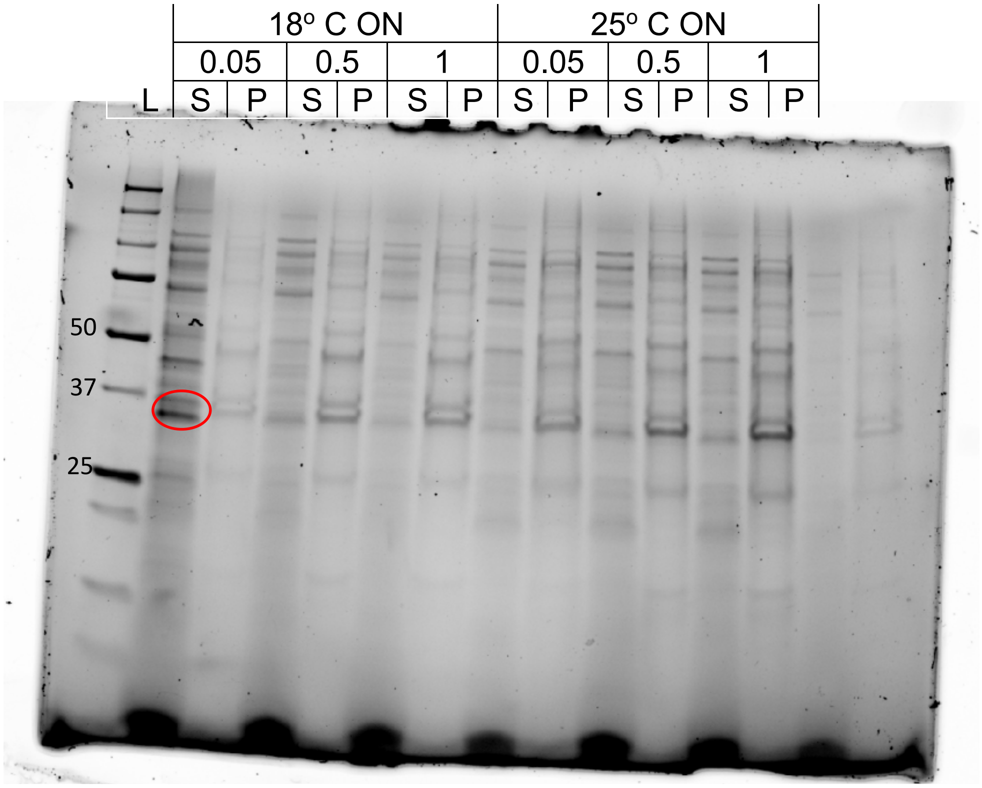

Supplement: S1 Fig — A, B) Raw SDS-PAGE for gels shown in Fig 1A and 1B; respectively, letter abbreviations are the same as in Fig 1 in text. (PDF) [file pone.0267226.s001.pdf]
