# Supplementary material for: Comparative expression of soluble, active human kinases in specialized bacterial strains
Source: PLoS One. 2022 Apr 19;17(4):e0267226. doi: 10.1371/journal.pone.0267226 (PMC9017934; doi:10.1371/journal.pone.0267226)

S2 Fig. Raw SDS-PAGE gels showing the effect of adding folding chaperones on expression A, B) Raw SDS-PAGE for gels shown in Figure 2A, and B; respectively, letter abbreviations are the same as in Figure 1 in text.

A.

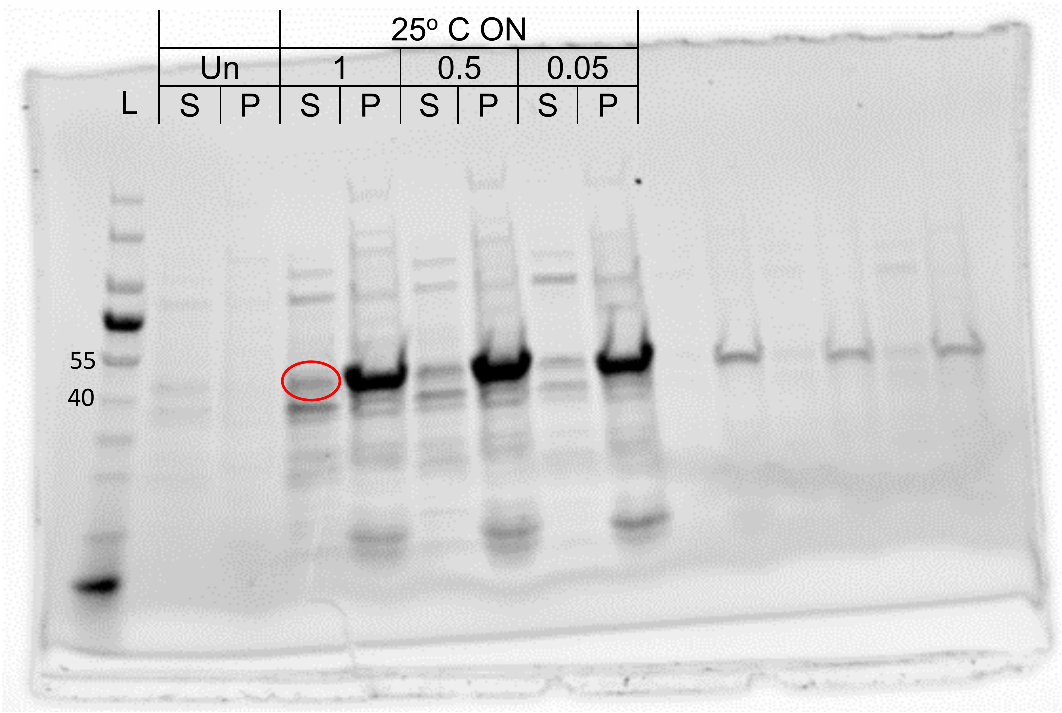

B.

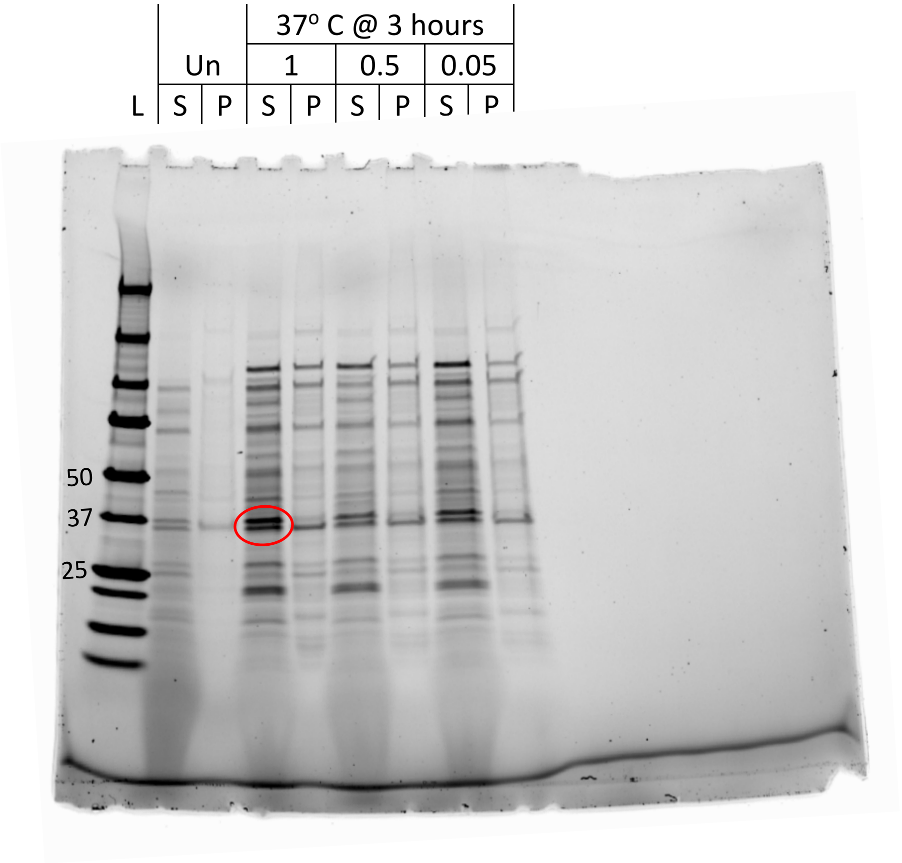

Supplement: S2 Fig — A, B) Raw SDS-PAGE for gels shown in Fig 2A and 2B; respectively, letter abbreviations are the same as in Fig 1 in text. (PDF) [file pone.0267226.s002.pdf]
