# Supplementary material for: Comparative expression of soluble, active human kinases in specialized bacterial strains
Source: PLoS One. 2022 Apr 19;17(4):e0267226. doi: 10.1371/journal.pone.0267226 (PMC9017934; doi:10.1371/journal.pone.0267226)

S3 Fig. Expression conditions for MKK3 A. Raw SDS-PAGE gel showing MKK3 expression in different strains and conditions that were difficult to compare across different initial expression gels, and B) Western blot with anti-His antibody for some of the samples to confirm protein identity, 2X refers to two-fold dilution of some samples to prevent overexposure and other letter abbreviations are the same as in Figure 1 in text.

A.

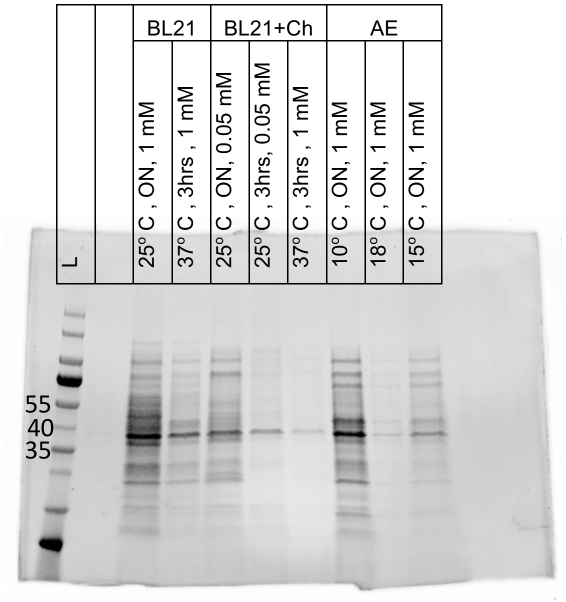

B.

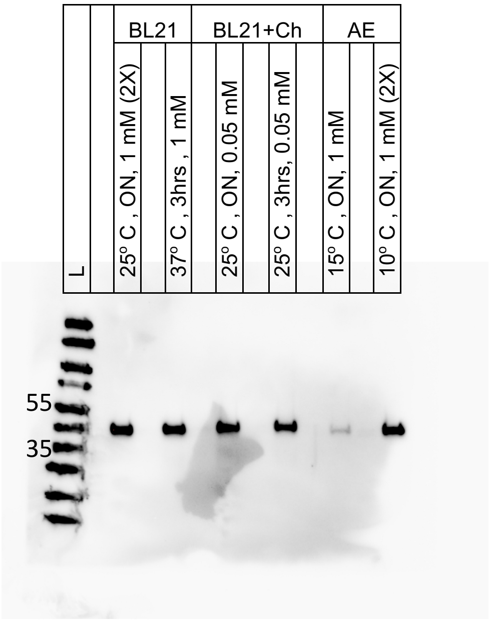

Supplement: S3 Fig — A. Raw SDS-PAGE gel showing MKK3 expression in different strains and conditions that were difficult to compare across different initial expression gels, and B) Western blot with anti-His antibody for some of the samples to confirm protein identity, 2X refers to two-fold dilution of some samples to prevent overexposure and other letter abbreviations are the same as in Fig 1 in text. (PDF) [file pone.0267226.s003.pdf]
