# Supplementary figures and images for: Comparative expression of soluble, active human kinases in specialized bacterial strains
Source: PLoS One. 2022 Apr 19;17(4):e0267226. doi: 10.1371/journal.pone.0267226 (PMC9017934; doi:10.1371/journal.pone.0267226)

**S4 Fig. Raw SDS-PAGE showing the final purified proteins.** Raw image for gel shown in Figure 3 in text.

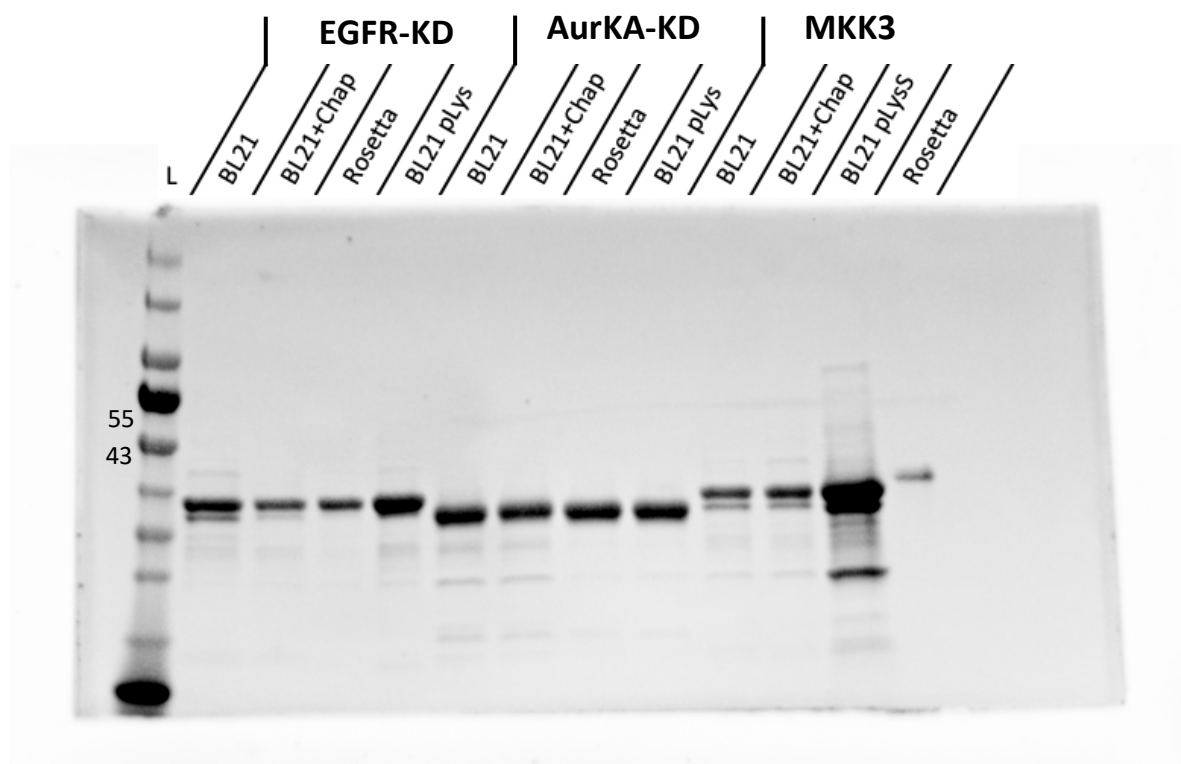

Supplement: S4 Fig — Raw image for gel shown in Fig 3 in text. (PDF) [file pone.0267226.s004.pdf]
