# Supplementary material for: Comparative expression of soluble, active human kinases in specialized bacterial strains
Source: PLoS One. 2022 Apr 19;17(4):e0267226. doi: 10.1371/journal.pone.0267226 (PMC9017934; doi:10.1371/journal.pone.0267226)

S5 Fig. SEC Chromatograms of A) EGFR-KD, B) AurKA-KD, and C) MKK3 in all strains. The inset show the twin peaks profile observed in the SEC profiles of the same constructs prepared in SF9 insect cells for EGFR-KD separated on the same SEC coumn (reproduced with permission from reference 19, Figure 4), and *pichia pastoris* for AurKA-KD and MKK3 separated on HiLoad 16/600 Superdex 200 pg (SPS-PAGE for the three shown profiles can be found in references 4 and 19).

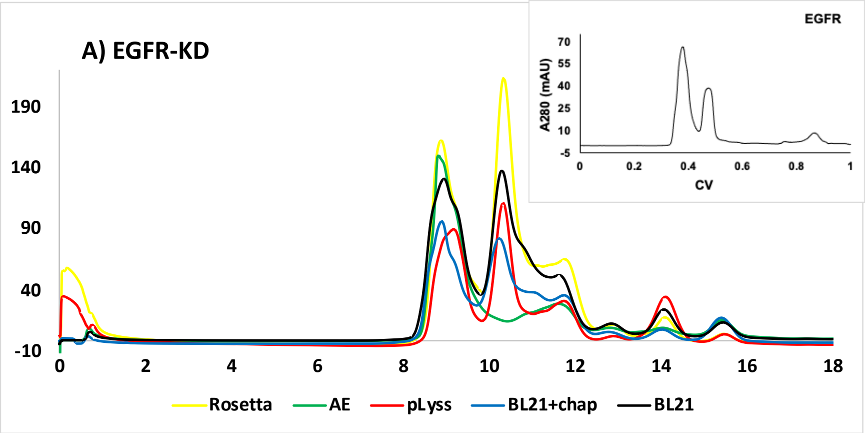

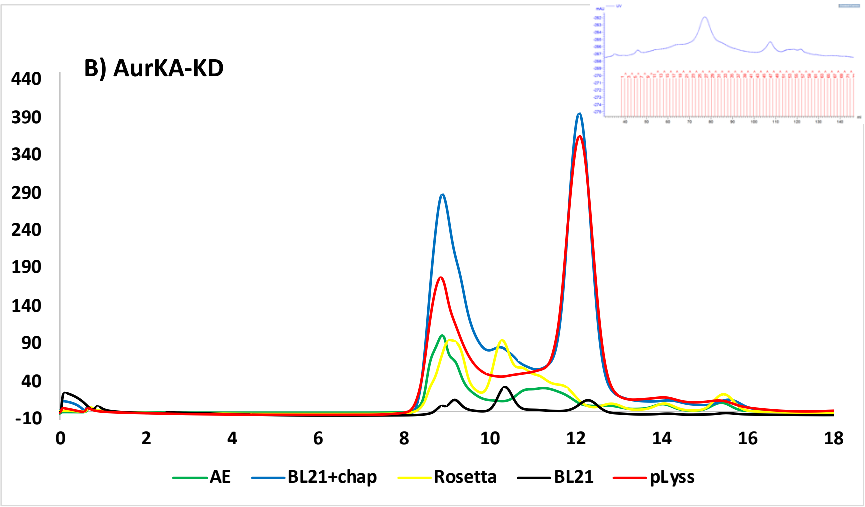

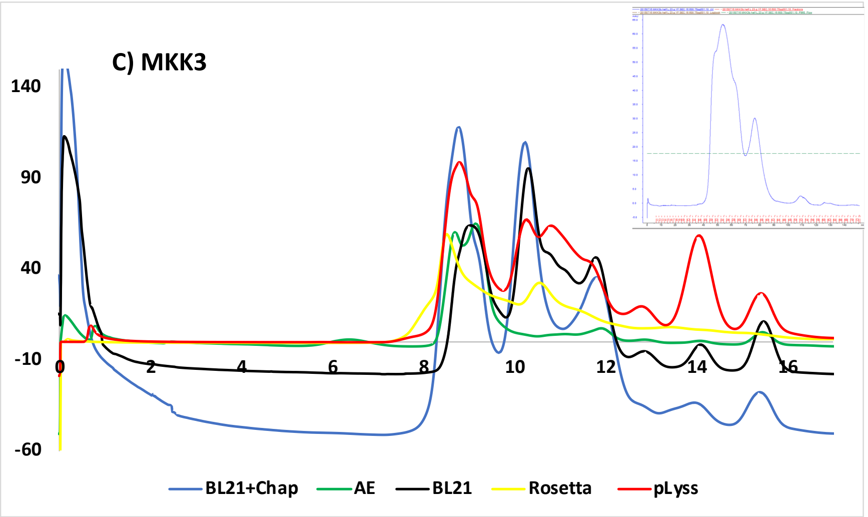

Supplement: S5 Fig — The inset show the twin peaks profile observed in the SEC profiles of the same constructs prepared in SF9 insect cells for EGFR-KD separated on the same SEC coumn (reproduced with permission from reference 19, Fig 4), and Pichia pastoris for AurKA-KD and MKK3 separated on HiLoad 16/600 Superdex 200 pg (SPS-PAGE for the three shown profiles can be found in references 4 and 19). (PDF) [file pone.0267226.s005.pdf]
