# Supplementary material for: Comparative expression of soluble, active human kinases in specialized bacterial strains
Source: PLoS One. 2022 Apr 19;17(4):e0267226. doi: 10.1371/journal.pone.0267226 (PMC9017934; doi:10.1371/journal.pone.0267226)

S6 Fig. Size distribution plot for EGFR-KD. The particle size profiles from samples expressed in the reported bacterial strains are compared to the same construct prepared in sf9 insect cells and characterized in reference 19.

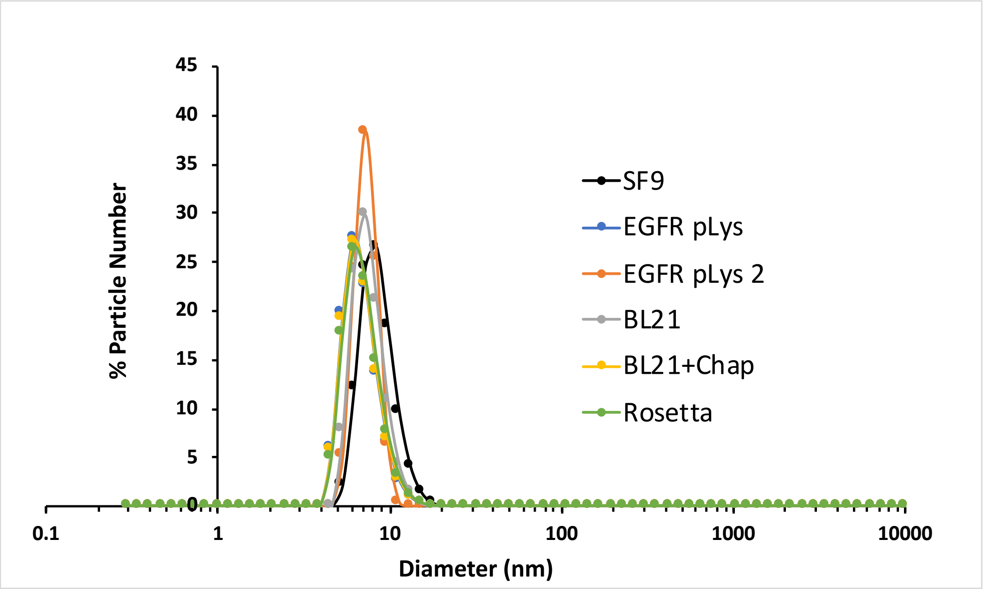

Supplement: S6 Fig — The particle size profiles from samples expressed in the reported bacterial strains are compared to the same construct prepared in sf9 insect cells and characterized in reference 19. (PDF) [file pone.0267226.s006.pdf]
