# Supplementary material for: Comparative expression of soluble, active human kinases in specialized bacterial strains
Source: PLoS One. 2022 Apr 19;17(4):e0267226. doi: 10.1371/journal.pone.0267226 (PMC9017934; doi:10.1371/journal.pone.0267226)

S7 Fig. Stability of the purified kinases at different Temperatures. The enzymes were stored at the indicated temperature and the activity was measured at the indicated time points as detailed in the Materials section.

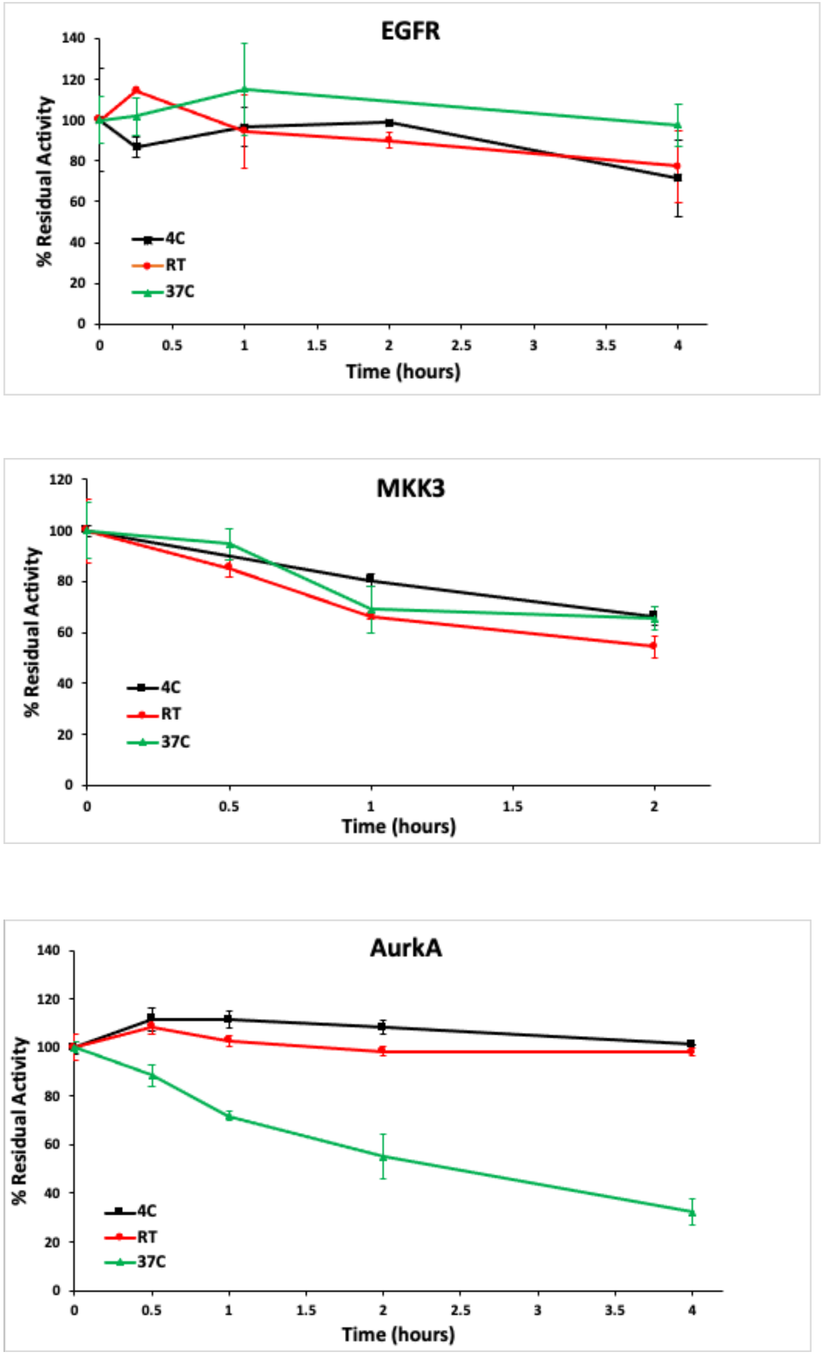

Supplement: S7 Fig — The enzymes were stored at the indicated temperature and the activity was measured at the indicated time points as detailed in the Materials section. (PDF) [file pone.0267226.s007.pdf]
