# Supplementary material for: Comparative expression of soluble, active human kinases in specialized bacterial strains
Source: PLoS One. 2022 Apr 19;17(4):e0267226. doi: 10.1371/journal.pone.0267226 (PMC9017934; doi:10.1371/journal.pone.0267226)
Supplement: S1 Table — The antibiotics used in culture were ampicillin at 100 μg/ml as the selection marker for the pET-15b plasmid used to host the kinases, in addition to the pre-existing resistance marker for each strain, if applicable. (PDF) [file pone.0267226.s008.pdf]

S1 Table. *E. coli* strains used in the study. The antibiotics used in culture were ampicillin at 100 µg/ml as the selection marker for the pET-15b plasmid used to host the kinases, in addition to the pre-existing resistance marker for each strain, if applicable.

	Genotype	Pre-existing resistance marker
DH5α	F– φ80lacZΔM15 Δ(lacZYA-argF)U169 recA1 endA1 hsdR17(rK–, mK+) phoA supE44 λ– thi-1 gyrA96 relA1	None
BL21 (DE3)	F^- *omp*T *hsd*S_B (r_B^-, m_B^-) *gal dcm* (DE3)	None
BL21 (DE3) pLysS	F^- *omp*T *hsd*S_B (r_B^-, m_B^-) *gal dcm* (DE3) pLysS(Cam^R)	Chloramphenicol
Rosetta (DE3)	F^- *ompT hsdS*$_B$(r_B^- m_B^-) *gal dcm* (DE3) pRARE (Cam^R)	Chloramphenicol
Arctic Express (DE3)	F^- ompT hsdS(r^- m^-) dcm^+ Tet^r gal λ(DE3) endA Hte [cpn10 BB cpn60 $Gent^r$]	Gentamycin
