# Supplementary material for: Comparative expression of soluble, active human kinases in specialized bacterial strains
Source: PLoS One. 2022 Apr 19;17(4):e0267226. doi: 10.1371/journal.pone.0267226 (PMC9017934; doi:10.1371/journal.pone.0267226)
Supplement: S2 Table — The optimum conditions chosen for large-scale protein expression for the kinases in each bacterial strain are highlighted in bold (summarized in Table 1 in text). (PDF) [file pone.0267226.s009.pdf]

S2 Table. The inducer concentration with the highest expression level under different induction temperatures and times. The optimum conditions chosen for large-scale protein expression for the kinases in each bacterial strain are highlighted in bold (summarized in Table 1 in text).

	Induction temperature (time)	Optimum IPTG concentration (mM)*		
		**EGFR-KD**	**AurKA-KD**	**MKK3**
**BL21 (DE3)**				
	37 (3 hrs)	N	0.05	N
	30 (3 hrs)	N	N	N
	25 (3 hrs)	N	N	N
	25 (ON)	**0.05**	**0.05**	**1**
	18 (ON)	E	0.05	N
**BL21 (DE3) pLysS**				
	37 (3 hrs)	N	N	N
	30 (3 hrs)	E	N	N
	25 (3 hrs)	E	N	N
	25 (ON)	**1**	N	**1**
	18 (ON)	E	**0.05**	1
**BL21 (DE3) + Chaperones**				
	37 (3 hrs)	N	1	1
	30 (3 hrs)	N	0.05	N
	25 (3 hrs)	0.5	1	0.05
	25 (ON)	**1**	**0.5**	**0.05**
	18 (ON)	0.5	1	N
**Rosetta**				
	37 (3 hrs)	0.5	0.05	N
	30 (3 hrs)	0.05	E	E
	25 (3 hrs)	0.05	1	N
	25 (ON)	**0.05**	**1**	1
	18 (ON)	0.05	1	**1**
**Arctic Express**				
	10 (ON)	0.05	**0.05**	**1**
	15 (ON)	**0.05**	E	E
	18 (ON)	0.05	E	E

*N: No detected expression at all IPTG concentrations tested, E: all IPTG concentrations show equally weak expression.
