# Supplementary material for: Comparative expression of soluble, active human kinases in specialized bacterial strains
Source: PLoS One. 2022 Apr 19;17(4):e0267226. doi: 10.1371/journal.pone.0267226 (PMC9017934; doi:10.1371/journal.pone.0267226)
Supplement: S3 Table — Samples were assessed using dynamic light scattering as mentioned in Discussion section. PDI was calculated by dividing the square of the standard deviation of the protein peak by the square of its average diameter. (PDF) [file pone.0267226.s010.pdf]

S3 Table. The average particle size diameter (d) and Polydispersity index (PDI) of the purified kinases. Samples were assessed using dynamic light scattering as mentioned in Discussion section. PDI was calculated by dividing the square of the standard deviation of the protein peak by the square of its average diameter.

	d (nm)	PDI
**EGFR-KD**		
BL21	8.69	0.104
BL21+Chap.	8.17	0.133
BL21 pLysS	7.47	0.104
Rosetta	8.17	0.133
**AurKa-KD**		
BL21	7.47	0.049
BL21+Chap.	7.69	0.166
BL21 pLysS	7.50	0.165
Rosetta	7.47	0.104
**MKK3**		
BL21	8.69	0.104
BL21+Chap.	8.49	0.023
BL21 pLysS	8.15	0.133
Rosetta	7.96	0.079
