# Supplementary material for: Comparative expression of soluble, active human kinases in specialized bacterial strains
Source: PLoS One. 2022 Apr 19;17(4):e0267226. doi: 10.1371/journal.pone.0267226 (PMC9017934; doi:10.1371/journal.pone.0267226)
Supplement: S4 Table — The activity was calculated by converting the rate of substrate formation in μmol/sec to nmol/min/mg enzyme and compared to the specific activity of commercially available active enzymes and/or the published results of the same constructs prepared in insect cells for EGFR-KD (reference 19 in text) or Pichia pastoris for AurKA-KD and MKK3 (reference 4 in text). (PDF) [file pone.0267226.s011.pdf]

S4 Table. The specific activity for each purified kinase. The activity was calculated by converting the rate of substrate formation in μmol/sec to nmol/min/mg enzyme and compared to the specific activity of commercially available active enzymes and/or the published results of the same constructs prepared in insect cells for EGFR-KD (reference 19 in text) or *pichia pastoris* for AurKA-KD and MKK3 (reference 4 in text).

**Specific Activity** (nmol/min/mg)	**Purified kinases**	**Reported**
**EGFR-KD**		
BL21	13.63 ± 2.70	
BL21+Chap.	21.27 ± 0.44	16.76 ± 0.64
BL21 pLysS	14.69 ± 3.00	31#
Rosetta	33.17 ± 4.08	
**AurKa-KD**		
BL21	47.69 ± 8.89	
BL21+Chap.	140.83 ± 8.93	18.7
BL21 plysS	130.24 ± 4.63	62 – 73^
Rosetta	15.88 ± 1.92	
**MKK3**		
BL21	9.76 ± 0.02	
BL21+Chap.	45.49 ± 0.95	32.6
BL21 plysS	11.57 ± 0.42	24 – 42^
Rosetta	73.52 ± 8.86	

The range of activity from most recent three lots if applicable for #His-tagged EGFR-KD and ^GST-tagged full-length AurKA and MKK3 expressed in sf9 insect cells as reported by SignalChem.
