# Supplementary material for: Comparative expression of soluble, active human kinases in specialized bacterial strains
Source: PLoS One. 2022 Apr 19;17(4):e0267226. doi: 10.1371/journal.pone.0267226 (PMC9017934; doi:10.1371/journal.pone.0267226)

<u>EGFR</u>

EGFR in BL21

Mw			25° C ON						18°C ON					
	Unind.		0.05		0.5		1		0.05		0.5		1	
	S	P	S	P	S	P	S	P	S	P	S	P	S	P

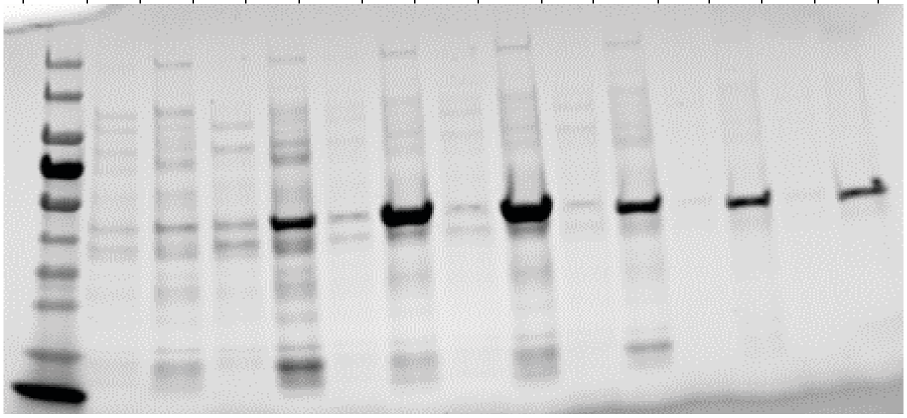

Mw			25° C @ 3 hours					
	Unind.		0.05		0.5		1	
	S	P	S	P	S	P	S	P

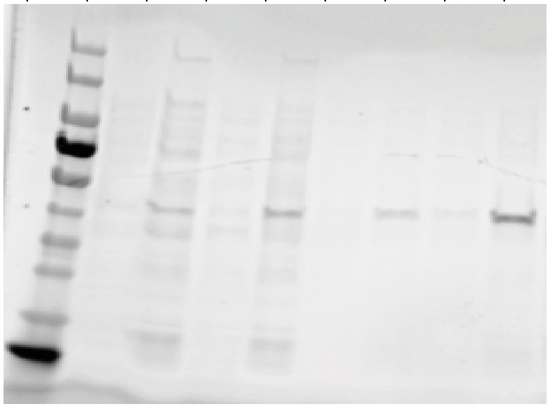

Mw			37° C @ 3 hours						30°C @ 3 hours					
	Unind.		0.05		0.5		1		0.05		0.5		1	
	S	P	S	P	S	P	S	P	S	P	S	P	S	P

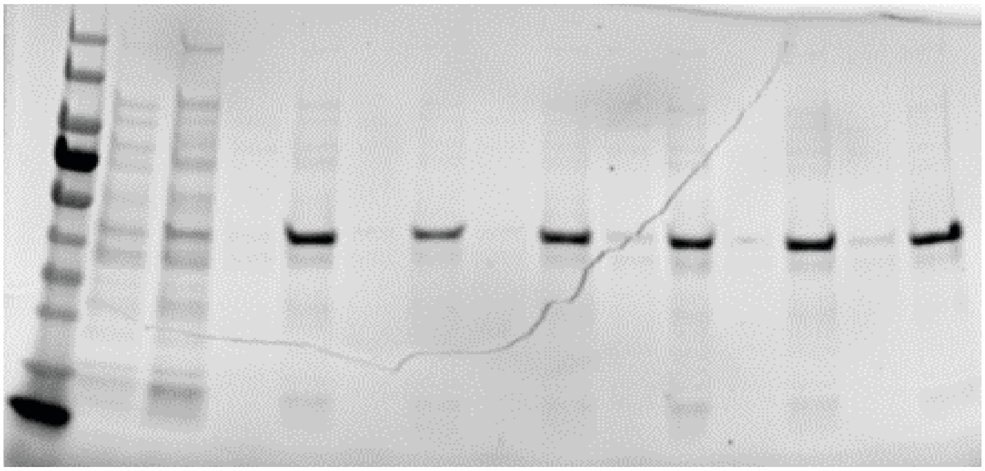

EGFR in BL21 plysS

			18° C ON						25°C ON					
	Unind.		1		0.5		0.05		1		0.5		0.05	
M_w	S	P	S	P	S	P	S	P	S	P	S	P	S	P

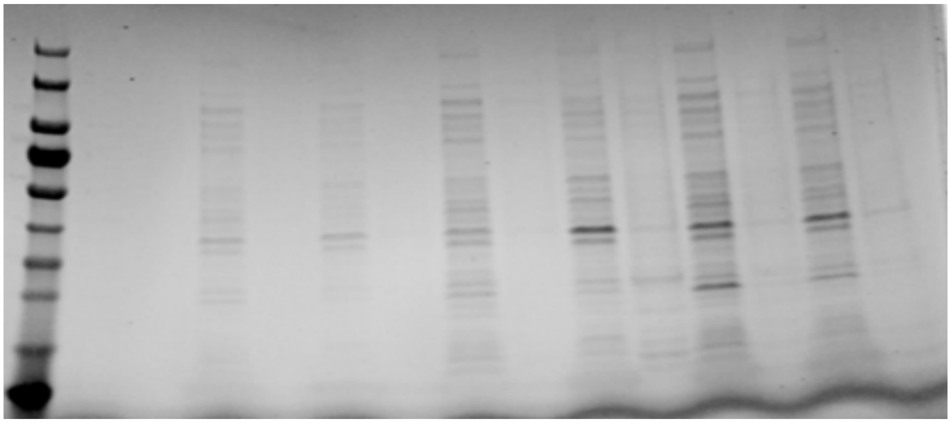

			25° C @ 3 hours						30°C @ 3 hours					
	Unind.		1		0.5		0.05		1		0.5		0.05	
M_w	S	P	S	P	S	P	S	P	S	P	S	P	S	P

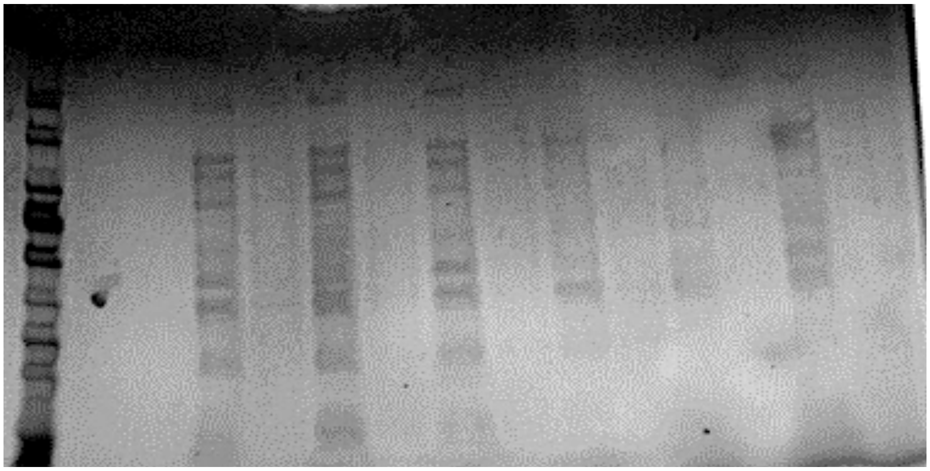

			37° C @ 3 hours					
	Unind.		1		0.5		0.05	
M_w	S	P	S	P	S	P	S	P

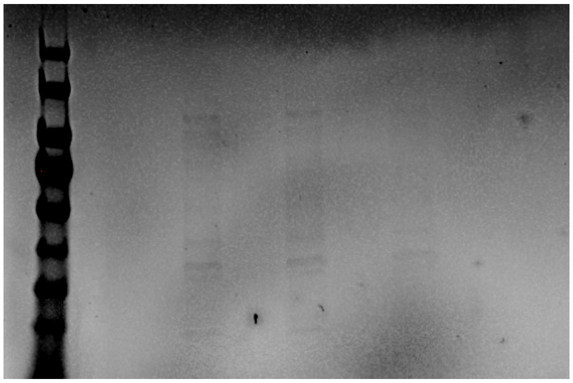

EGFR in Rosetta

A

			18°C ON						25°C @ 3 hours					
	Unind.		0.05		0.5		1		0.05		0.5		1	
M_w	S	P	S	P	S	P	S	P	S	P	S	P	S	P	S	P

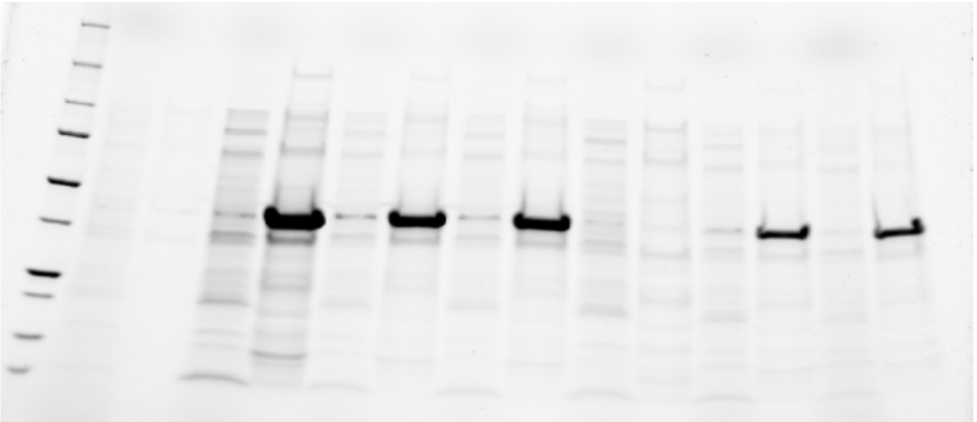

			25°C ON						30°C @ 3 hours					
	Unind.		0.05		0.5		1		0.05		0.5		1	
M_w	S	P	S	P	S	P	S	P	S	P	S	P	S	P	S	P

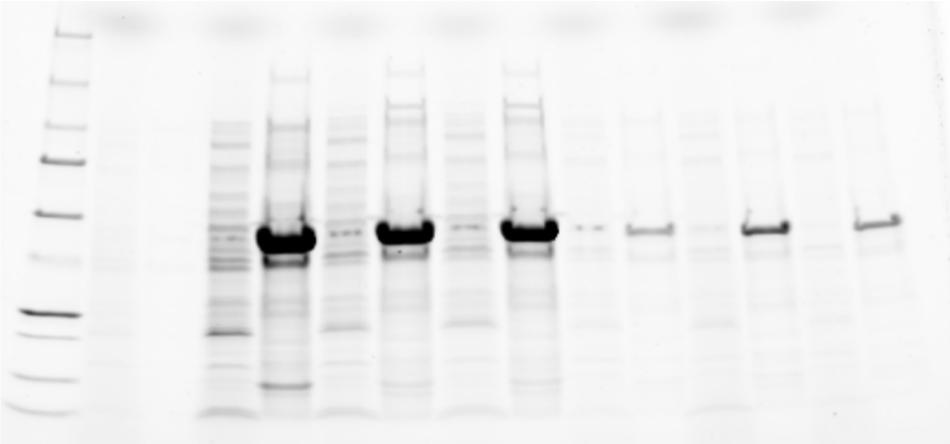

			37°C @ 3 hours					
	Unind.		0.05		0.5		1	
M_w	S	P	S	P	S	P	S	P

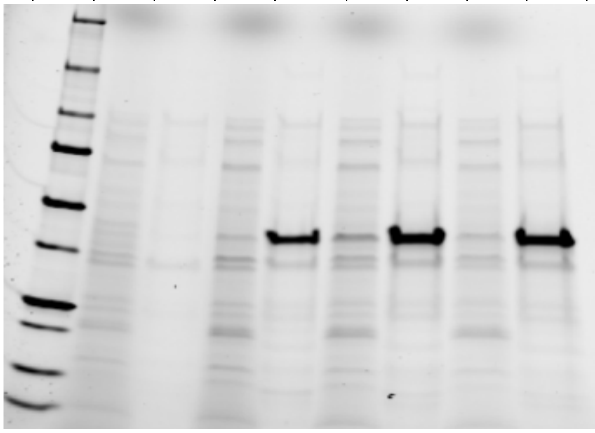

EGFR in BL21+Chap.

A

			18° C ON						25°C @ 3 hours					
	Unind.		1		0.5		0.05		1		0.5		0.05	
Mw	S	P	S	P	S	P	S	P	S	P	S	P	S	P

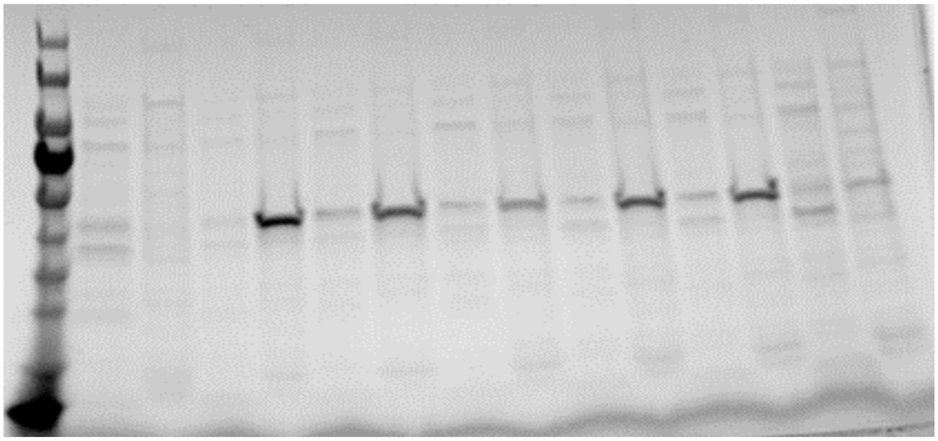

			25° C ON						30°C @ 3 hours					
	Unind.		1		0.5		0.05		1		0.5		0.05	
Mw	S	P	S	P	S	P	S	P	S	P	S	P	S	P

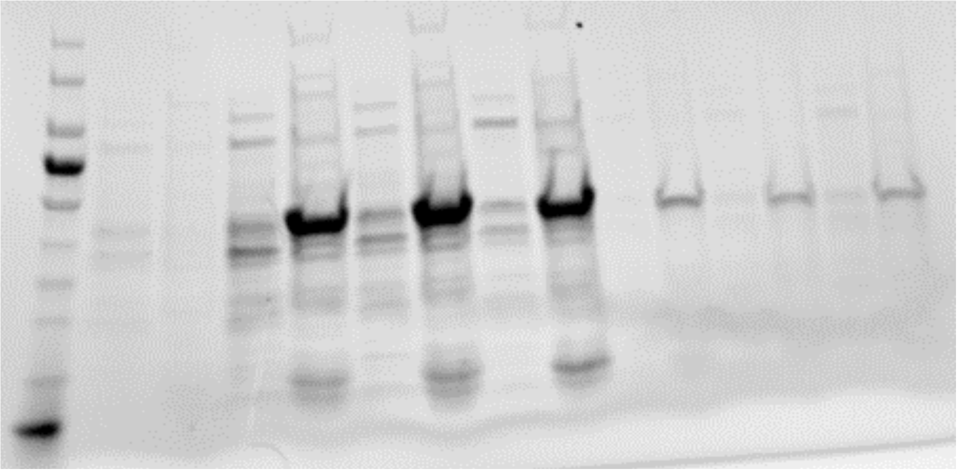

			37°C @ 3 hours					
	Unind.				0.5		0.05	
Mw	S	P	S	P	S	P	S	P

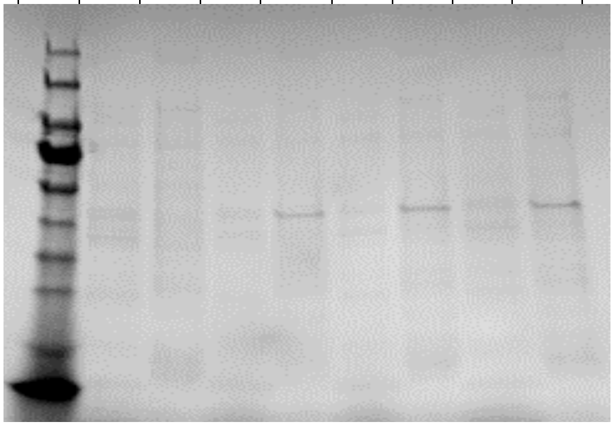

EGFR in Arctic Express

A

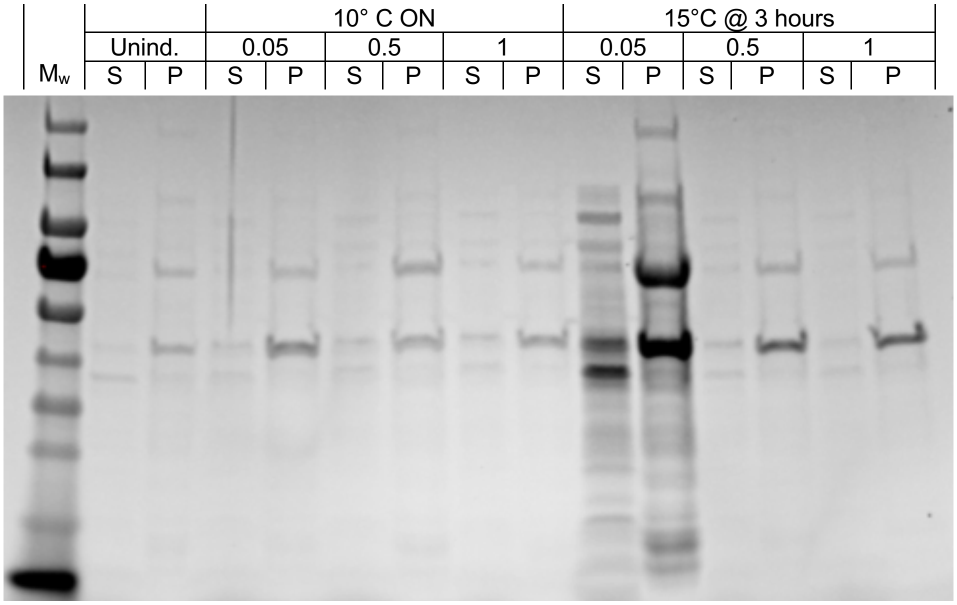

B

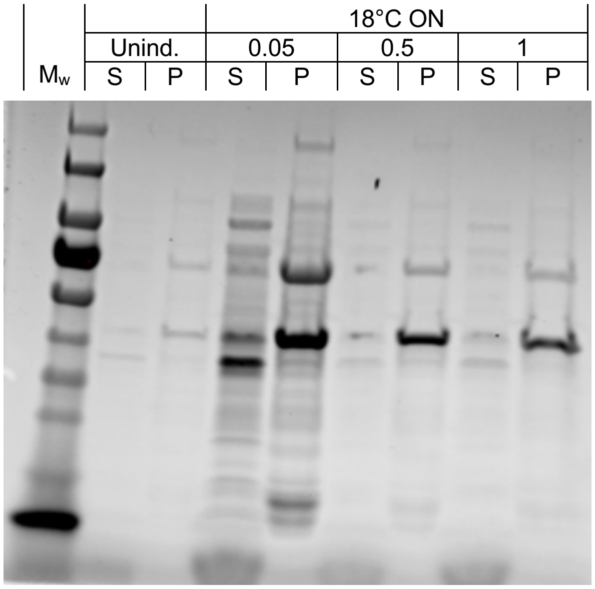

EGFR His Trap FF 1L purification

EGFR BL21 1L

M_w | L | F | E

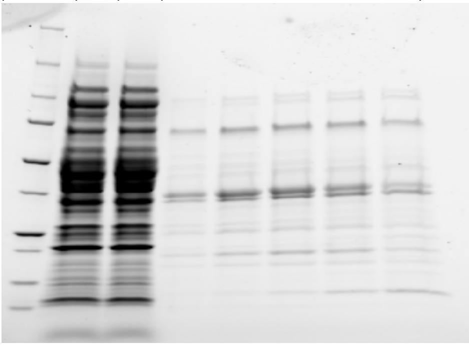

EGFR BL21 plysS 1L

M_w | L | F | E

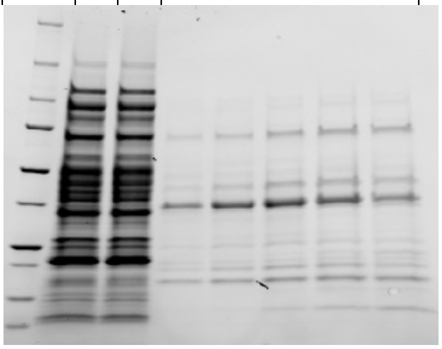

EGFR Rosetta

M_w | L | F | E

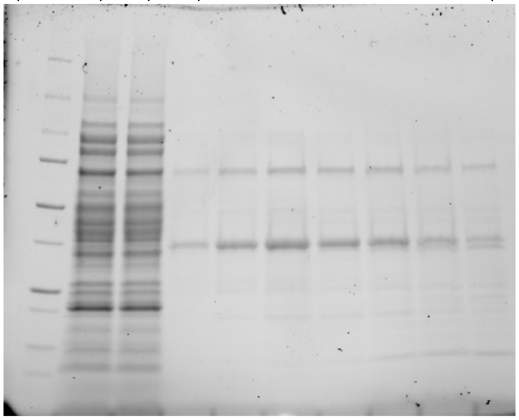

EGFR BL21 with chaperone

M_w | L | F | E

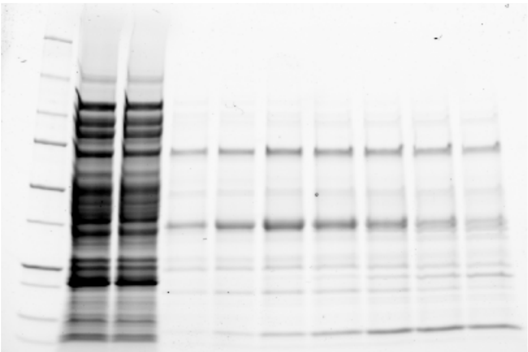

EGFR Arctic Express

M_w | L | F | E

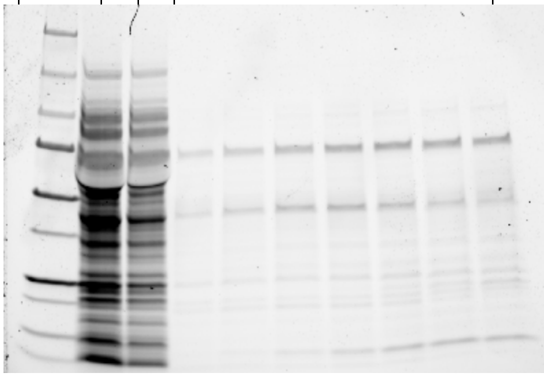

AurkA

AurkA in BL21

Mw	Unind.		18° C ON						25° C @ 3 hours	
			1		0.5		0.05		1	
	S	P	S	P	S	P	S	P	S	P

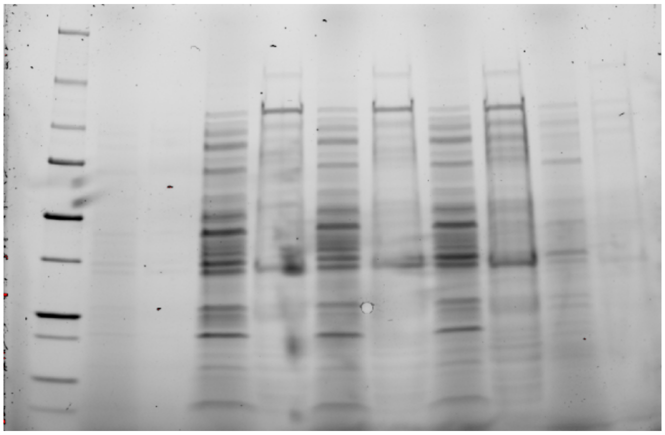

Mw	Unind.		25° C @ 3 hours				25° C ON			
			0.5		0.05		1		0.5	
	S	P	S	P	S	P	S	P	S	P

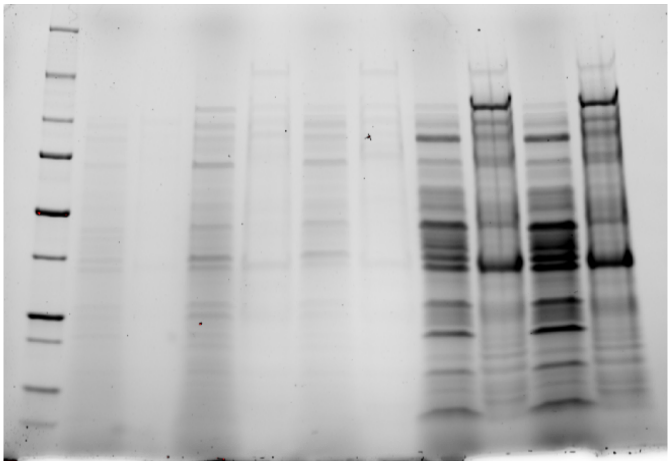

Mw	Unind.		25° C ON		30° C @ 3 hours					
			0.05		1		0.5		0.05	
	S	P	S	P	S	P	S	P	S	P

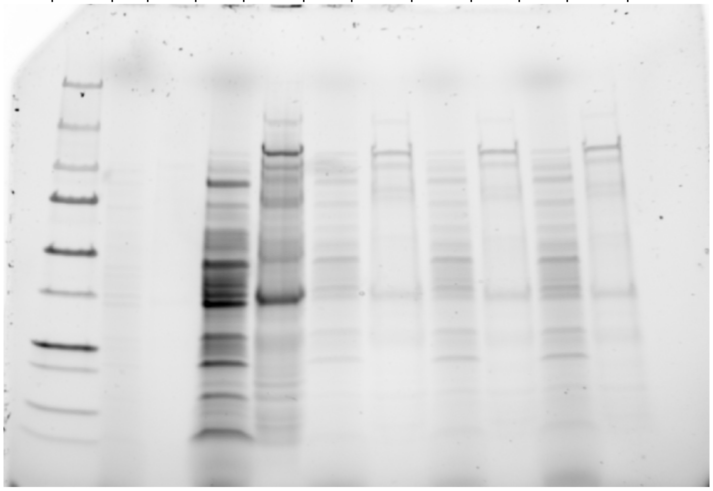

Mw	37° C @ 3 hours					
	0.05		0.5		1	
	S	P	S	P	S	P

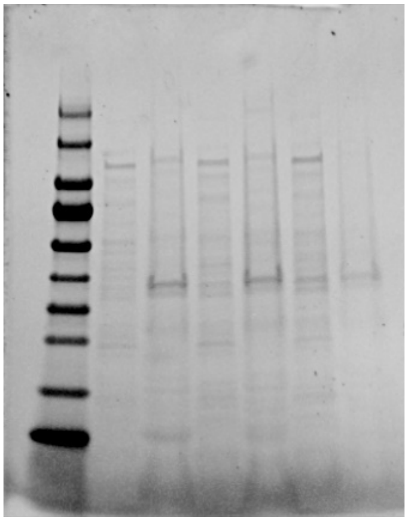

AurkA in BL21 pLysS

	18° C ON						25° C ON					
	0.05		0.5		1		0.05		0.5		1	
M_w	S	P	S	P	S	P	S	P	S	P	S	P

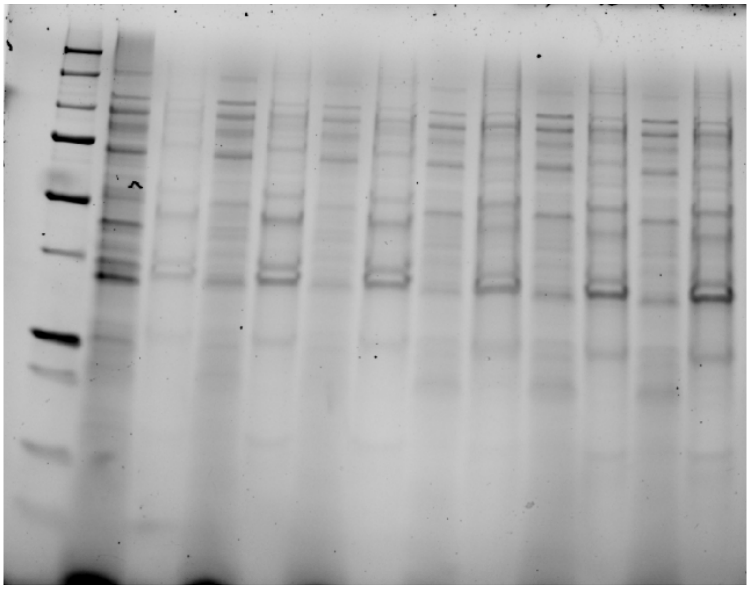

	25° C @ 3 hours						30° @ 3 hours					
	0.05		0.5		1		0.05		0.5		1	
M_w	S	P	S	P	S	P	S	P	S	P	S	P

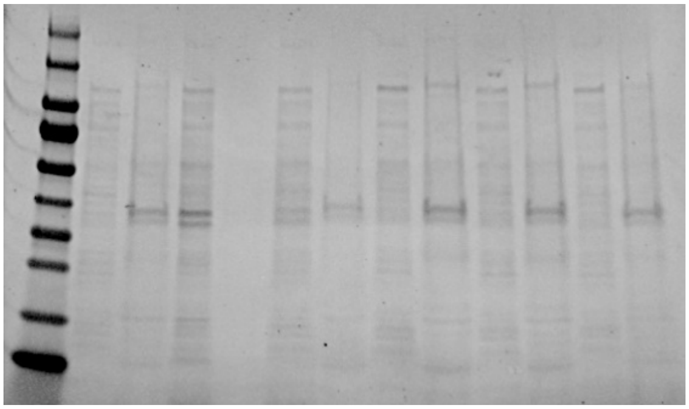

			37° C @ 3 hours					
	Unind.		1		0.5		0.05	
M_w	S	P	S	P	S	P	S	P

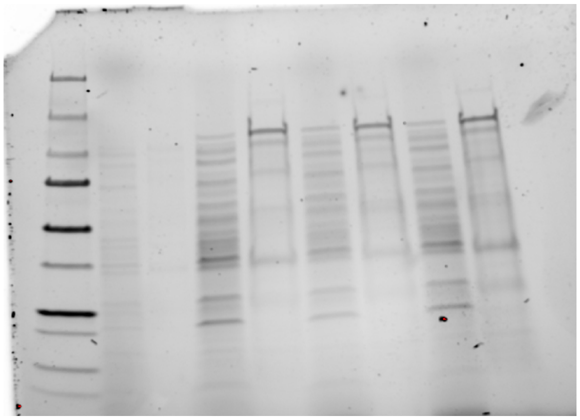

AurKA in Rosetta

			25° C @ 3 hours						30° C @ 3 hours					
	Unind		1		0.5		0.05		1		0.5		0.05	
M_w	S	P	S	P	S	P	S	P	S	P	S	P	S	P

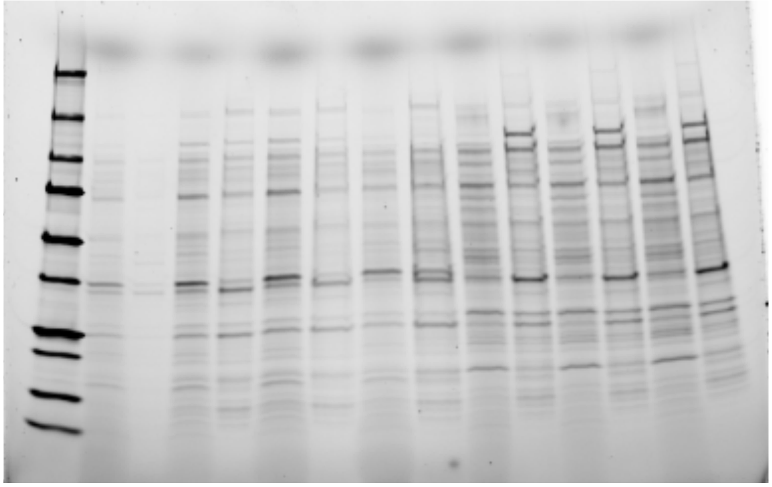

37° C @ 3 hours					
1		0.5		0.05	
S	P	S	P	S	P

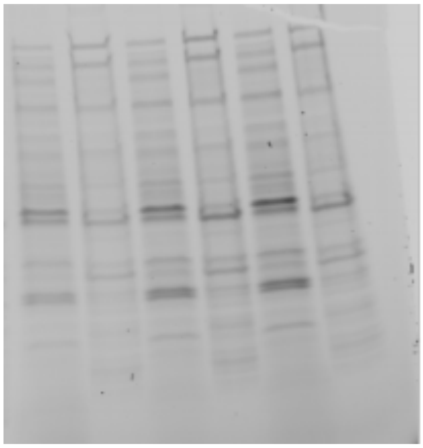

AurKA in BL21+Chap

			18° C ON						25° C ON					
	Unind.		1		0.5		0.05		1		0.5		0.05	
Mw	S	P	S	P	S	P	S	P	S	P	S	P	S	P

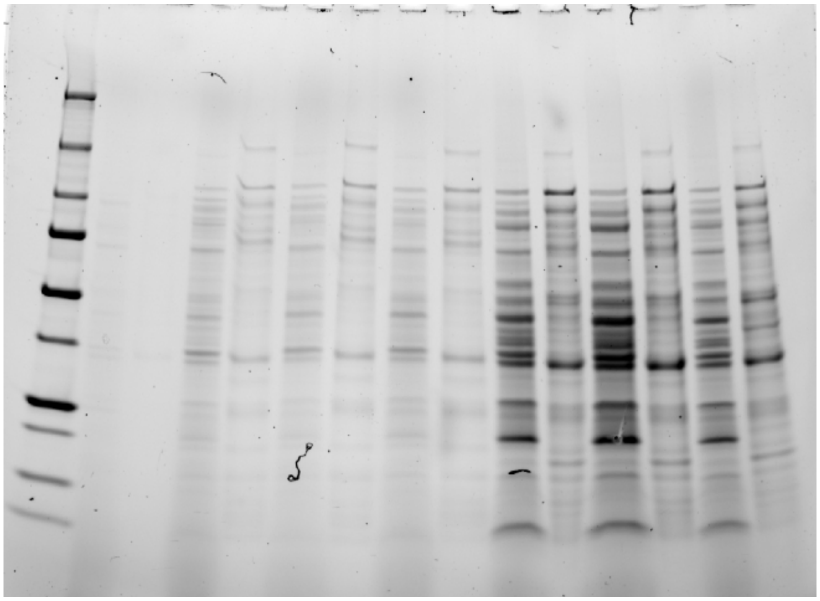

			25° C @ 3 hours						30° C @ 3 hours					
	Unind.		1		0.5		0.05		1		0.5		0.05	
Mw	S	P	S	P	S	P	S	P	S	P	S	P	S	P

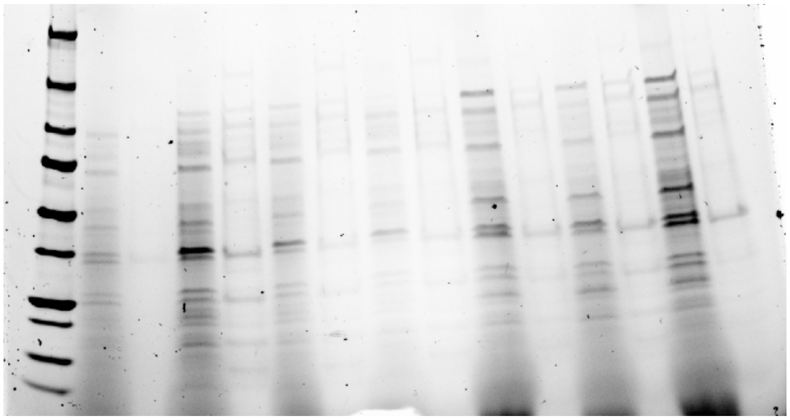

			37° C @ 3 hours					
	Unind.		1		0.5		0.05	
Mw	S	P	S	P	S	P	S	P

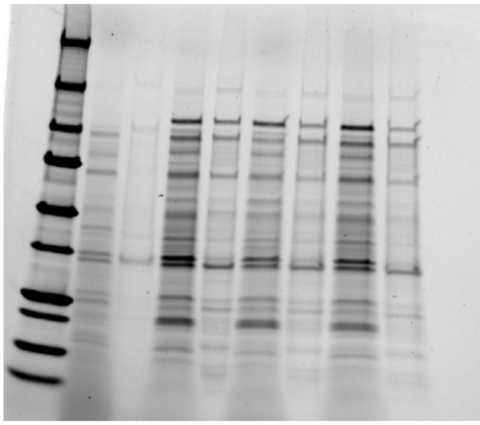

AurKA in Arctic Express

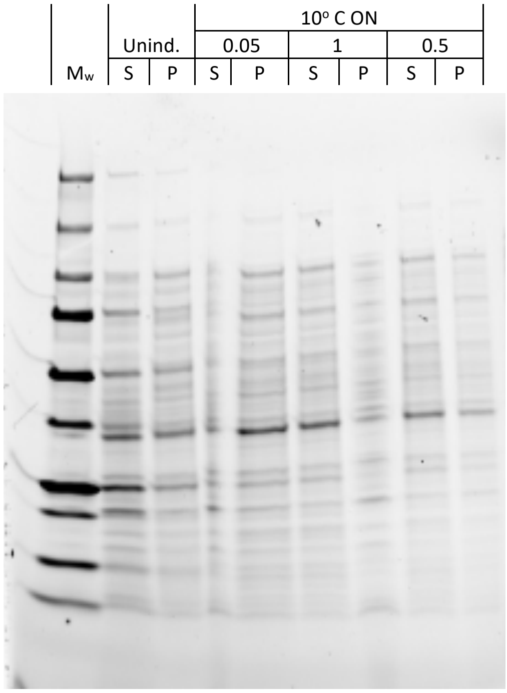

AurKA His Trap FF 1L purification

BL21 1L Purification

M_w | L | F | E

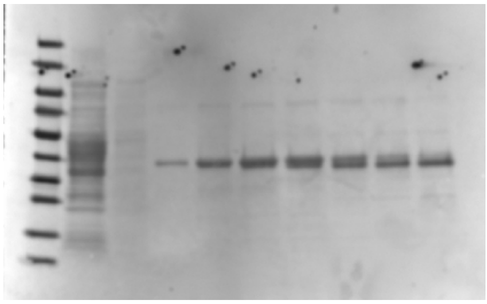

BL21 plysS 1L Purification

M_w | L | F | E

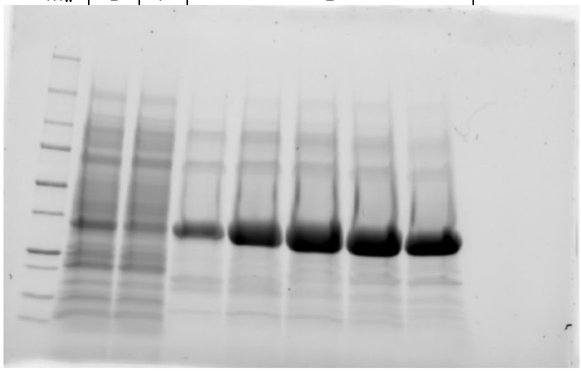

Rosetta 1L Purification

M_w | L | W | E

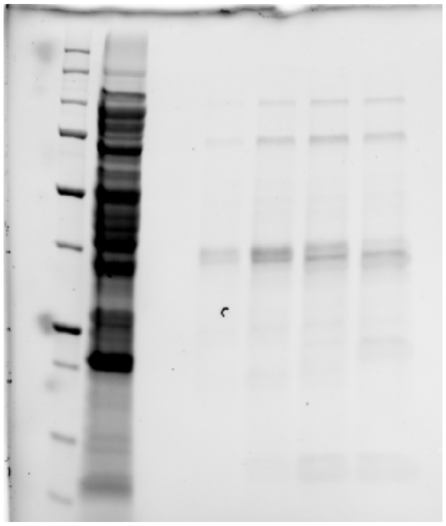

BL21 with Chaperone 1L Purification

M_w | L | F | E

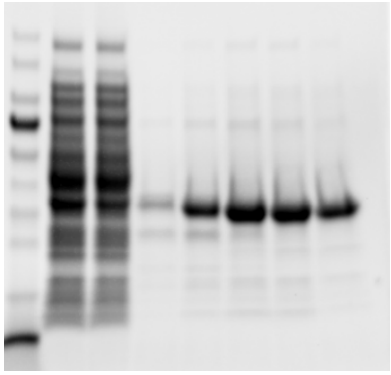

Arctic Express IL Purification

M_w | L | F | E

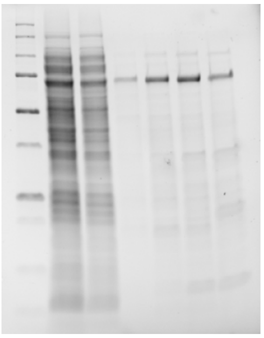

MKK3

MKK3 in BL21’

			18° C ON						25° C ON					
	Unind.		1		0.5		0.05		1		0.5		0.05	
M_w	S	P	S	P	S	P	S	P	S	P	S	P	S	P

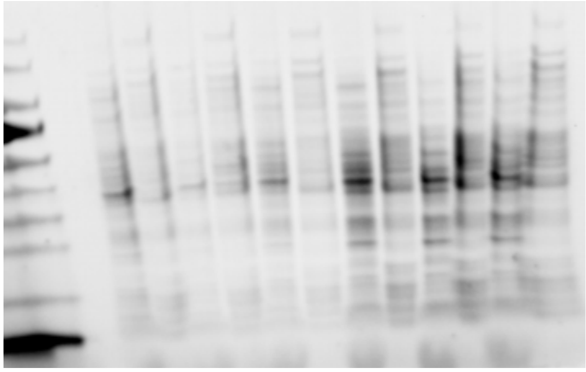

			30° C @ 3 hours						25° C @ 3 hours					
	Unind		1		0.5		0.05		1		0.5		0.05	
M_w	S	P	S	P	S	P	S	P	S	P	S	P	S	P

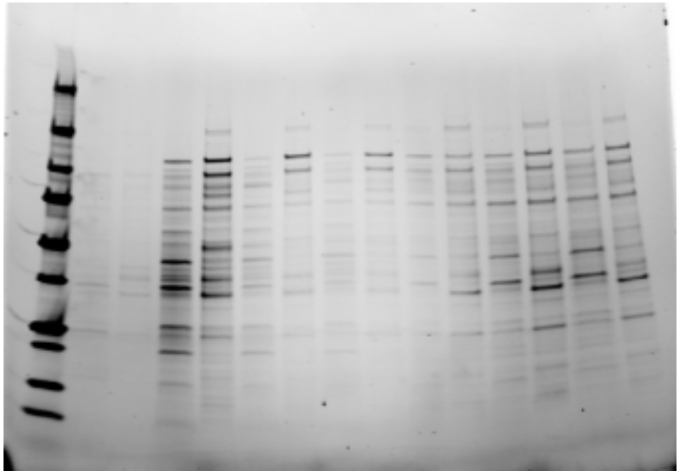

			37° C @ 3 hours					
	Unind.		1		0.5		0.05	
M_w	S	P	S	P	S	P	S	P

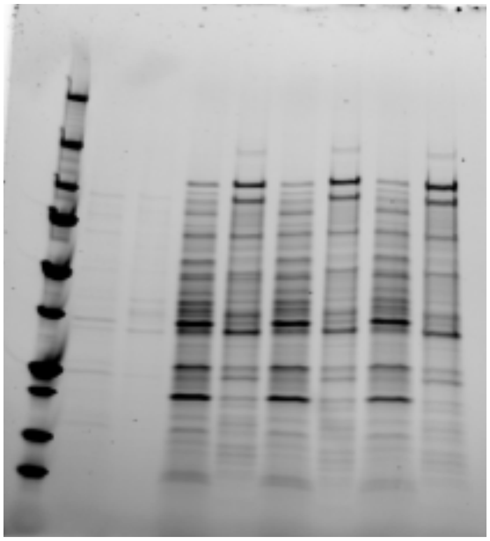

MKK3 in BL21 pLys

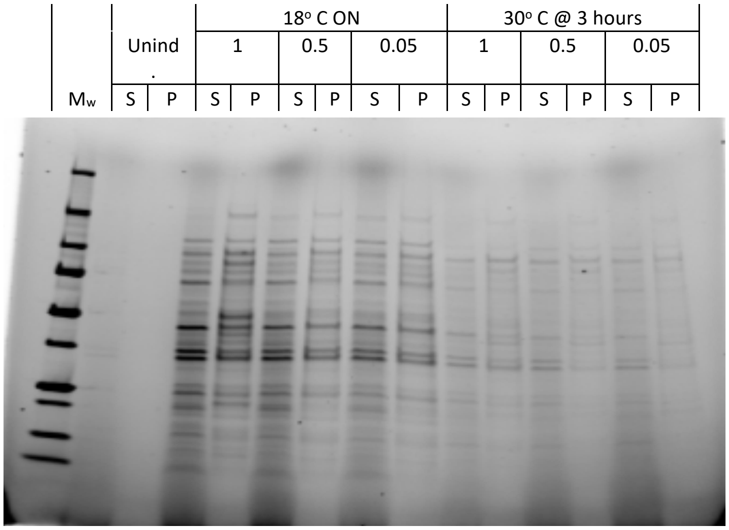

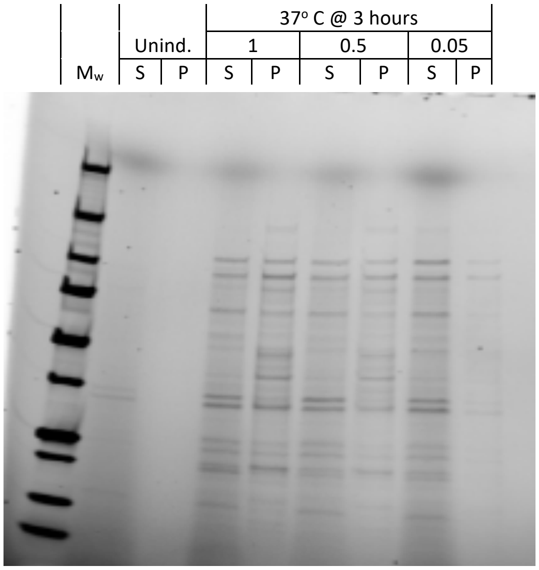

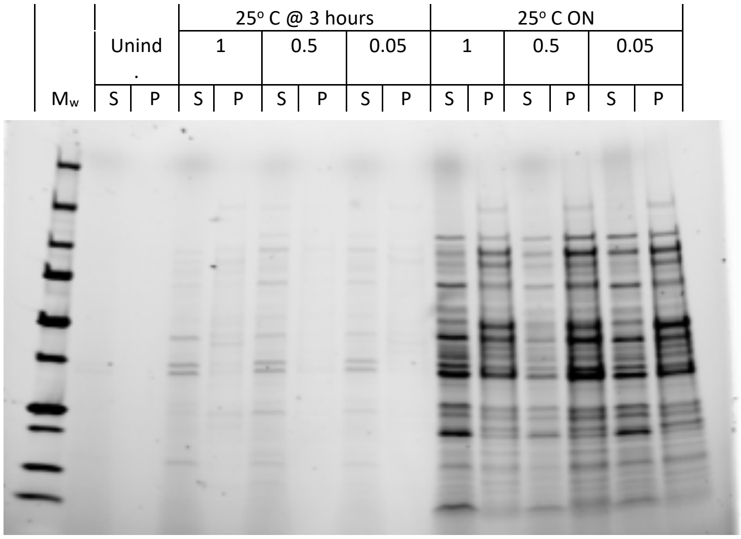

MKK3 in Rosetta

			18° C ON						25° C @ 3 hours					
	Unind.		1		0.5		0.05		1		0.5		0.05	
M_w	S	P	S	P	S	P	S	P	S	P	S	P	S	P

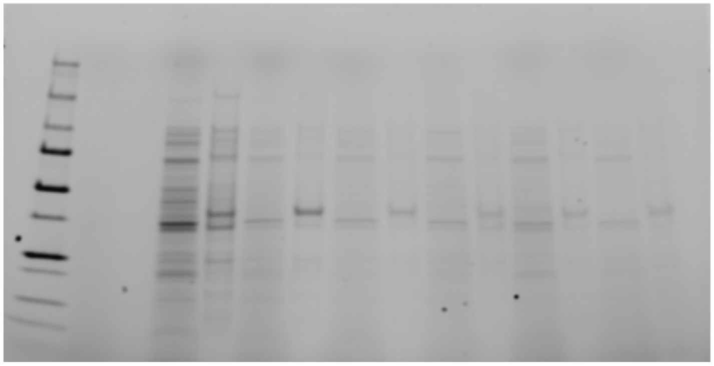

			30° C @ 3 hours					
	Unind.		1		0.5		0.05	
M_w	S	P	S	P	S	P	S	P

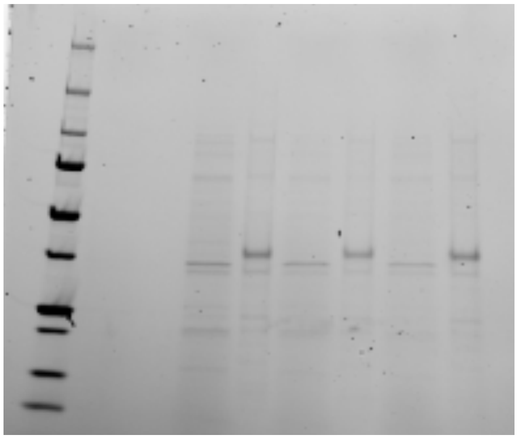

			25° C ON						37° C @ 3 hours					
	Unind		1		0.5		0.05		1		0.5		0.05	
M	S	P	S	P	S	P	S	P	S	P	S	P	S	P

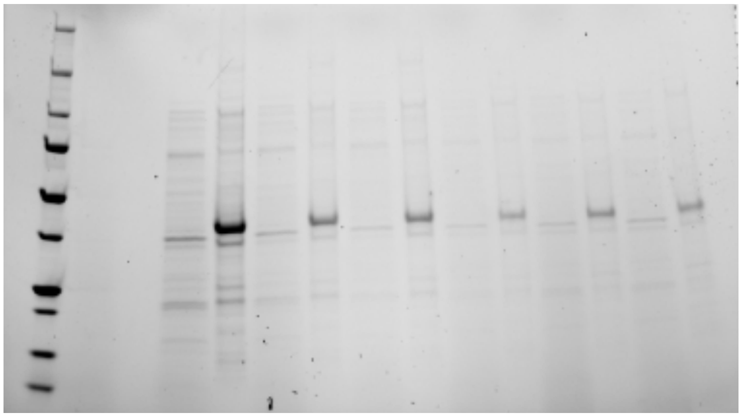

MKK3 in BL21+Chap

			25° C ON						18° C ON					
	Unind.		1		0.5		0.05		1		0.5		0.05	
Mw	S	P	S	P	S	P	S	P	S	P	S	P	S	P

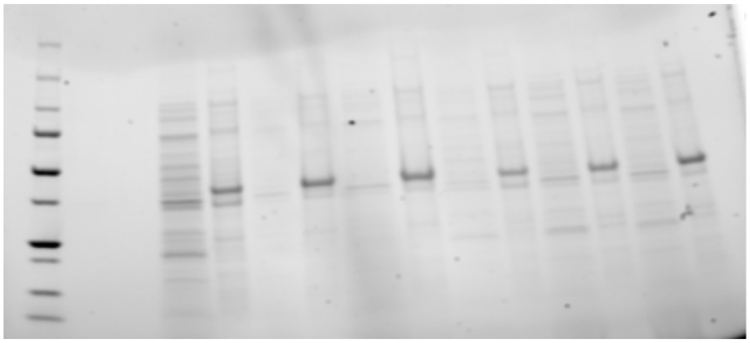

			25° C @ 3 hours						30° C @ 3 hours					
	Unind		1		0.5		0.05		1		0.5		0.05	
Mw	S	P	S	P	S	P	S	P	S	P	S	P	S	P

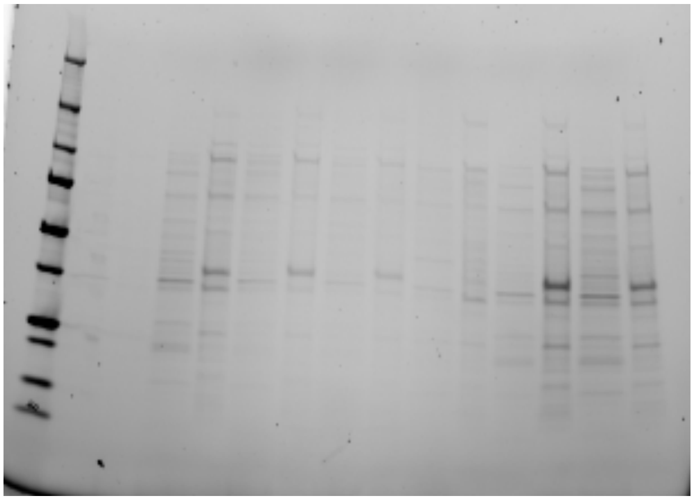

			37° C @ 3 hours					
	Unind.		1		0.5		0.05	
Mw	S	P	S	P	S	P	S	P

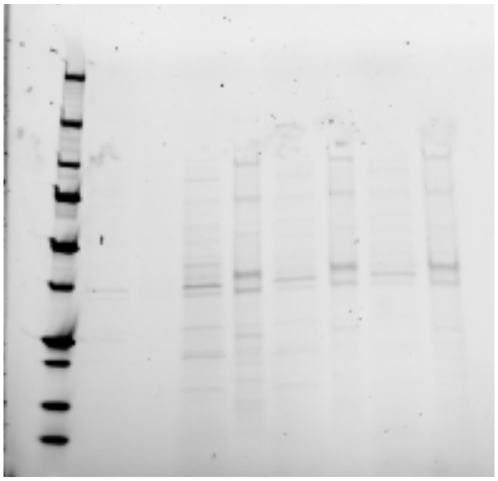

MKK3 in Arctic Express

			10° C ON					
	Unind.		1		0.5		0.05	
Mw	S	P	S	P	S	P	S	P

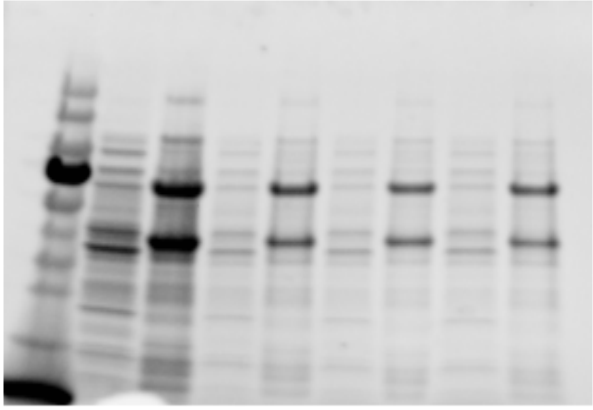

			18° C ON						15° C ON					
	Unind.		1		0.5		0.05		1		0.5		0.05	
Mw	S	P	S	P	S	P	S	P	S	P	S	P	S	P

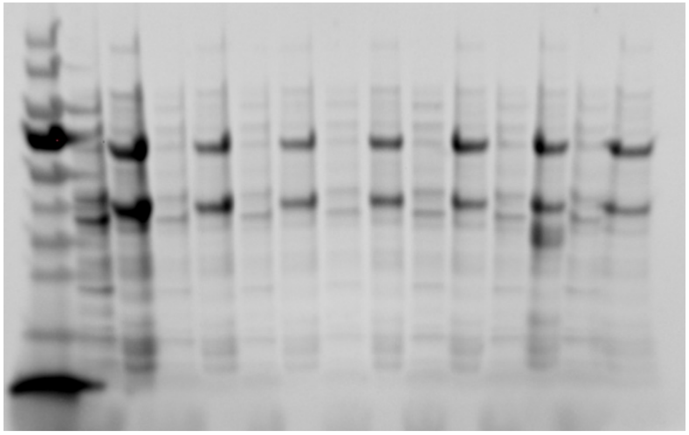

MKK3 His Trap FF 1L purification

Rosetta 1L Purification

| Mw | L | F | E |

BL21 1L Purification

| Mw | L | F | E |

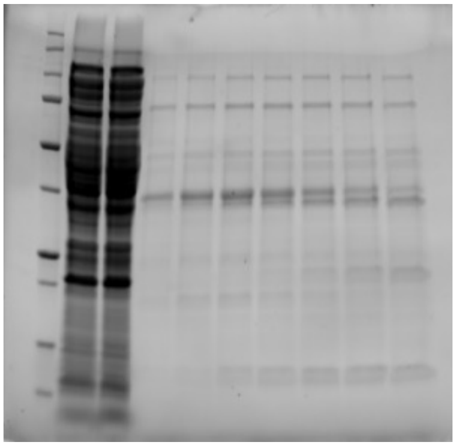

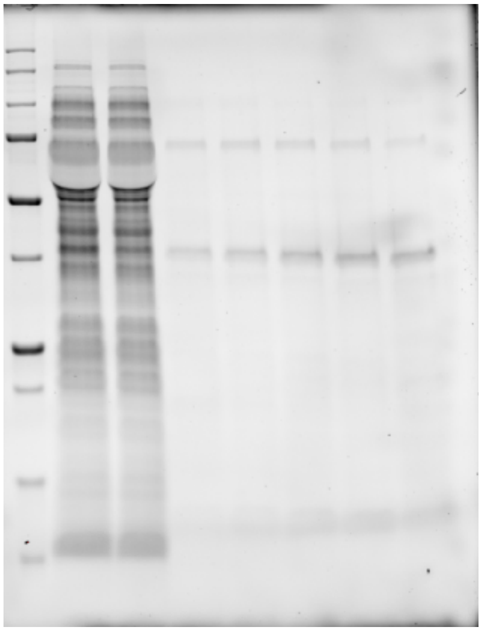

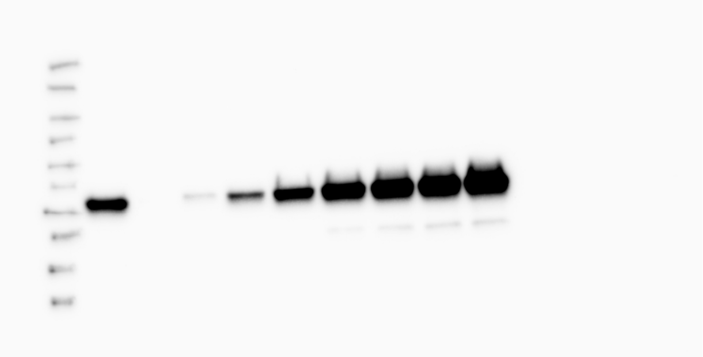

Supplement: S1 File — (PDF) [file pone.0267226.s012.pdf]
